# CircLONP2 enhances colorectal carcinoma invasion and metastasis through modulating the maturation and exosomal dissemination of microRNA-17

**DOI:** 10.1186/s12943-020-01184-8

**Published:** 2020-03-18

**Authors:** Kai Han, Feng-Wei Wang, Chen-Hui Cao, Han Ling, Jie-Wei Chen, Ri-Xin Chen, Zi-Hao Feng, Jie Luo, Xiao-Han Jin, Jin-Ling Duan, Shu-Man Li, Ning-Fang Ma, Jing-Ping Yun, Xin-Yuan Guan, Zhi-Zhong Pan, Ping Lan, Rui-Hua Xu, Dan Xie

**Affiliations:** 1 Sun Yat-sen University Cancer Center; State Key Laboratory of Oncology in South China, Collaborative Innovation Center for Cancer Medicine, Guangzhou, China; 2 Department of Colorectal Surgery, Sun Yat-sen University Cancer Center, Guangzhou, China; 3 Department of Pathology, Sun Yat-sen University Cancer Center, Guangzhou, China; 4 Department of Surgery, First Affiliated Hospital, Sun Yat-sen University, Guangzhou, China; 5 Key Laboratory of Protein Modification and Degradation, School of Basic Medical Sciences, Affiliated Cancer Hospital & Institute of Guangzhou Medical University, Guangzhou, China; 6 Department of Clinical Oncology, the University of Hong Kong, Hong Kong, China; 7 Department of Colorectal Surgery, the Six Affiliated Hospital, Sun Yat-sen University, Guangzhou, China

**Keywords:** Colorectal carcinoma, circLONP2, Metastasis, microRNA-17

## Abstract

**Background:**

Metastasis causes the vast majority of colorectal carcinoma (CRC)-related deaths. However, little is known about the specific traits and underlying mechanisms of metastasis-initiating cells in primary CRC. And whether or not circular RNAs (circRNAs) take part in this particular event remain not adequately stated yet.

**Methods:**

A screening method based on Transwell assay was first applied to build CRC subgroups with different metastatic potential. High throughput RNA sequencing was used to find out novel metastatic drivers in CRC metastasis-initiating step. A series of in vitro and in vivo assays were further applied to elucidate the functions and underlying molecular mechanisms of circRNAs in CRC metastasis.

**Results:**

A circRNA consisting of exon 8–11 of LONP2, termed as circLONP2, was upregulated in metastasis-initiating CRC subgroups. Aberrant higher expression of circLONP2 was observed in primary CRC tissues with established metastasis, and along the invasive margin in metastatic site. High expression of circLONP2 predicted unfavorable overall survival. Functional studies revealed that circLONP2 could enhance the invasiveness of CRC cells in vitro, and targeting circLONP2 through anti-sense oligonucleotide (ASO) dramatically reduced the penetrance of metastasis to foreign organs in vivo*.* Mechanically, circLONP2 directly interacted with and promoted the processing of primary microRNA-17 (pri-miR-17), through recruiting DiGeorge syndrome critical region gene 8 (DGCR8) and Drosha complex in DDX1-dependent manner. Meanwhile, upregulated mature miR-17-5p could be assembled into exosomes and internalized by neighboring cells to enhance their aggressiveness.

**Conclusions:**

Our data indicate that circLONP2 acts as key metastasis-initiating molecule during CRC progression through modulating the intracellular maturation and intercellular transfer of miR-17, resulting in dissemination of metastasis-initiating ability in primary site and acceleration of metastasis formation in foreign organs. circLONP2 could serve as an effective prognostic predictor and/or novel anti-metastasis therapeutic target in CRC treatment.

## Background

Colorectal carcinoma (CRC) ranks the third most common malignancy and the second leading cause of cancer-related deaths worldwide [1]. Although the therapeutic effect has been significantly improved during the last decades, metastasis remains the largest single cause of mortality and the most difficult challenge for CRC treatment. Metastasis is an important hallmark of cancer [2] and generally considered to be a multi-step procedure, during which cancer cells must go through the following steps: invasion, intravasation, survival in blood, extravasation and colonization at the metastatic site [3]. Invasion, a process that cancer cells migrate away from the primary tumor, is the first and most important step in metastasis formation [4]. Targeting those cancer cells with high invasion/metastasis-initiating potential from the primary site may be a promising field in anti-metastasis treatment. However, little is known about which subgroups of cancer cells initiate the invasion process and how they achieve that ability during CRC progression. This drawback has impeded the precise identification of CRC patients who may suffer high metastatic risks, as well as the therapeutic effects of anti-metastasis treatment.

Noncoding RNAs (ncRNAa) occupy most of the transcriptome, while only 2% of the genome can be transcribed into messenger RNAs (mRNAs) that would be translated into proteins [5]. Among all the subtypes of ncRNAs, circular RNAs (circRNAs), with particular circular form and once thought to be “rubbish” or “byproducts” of transcription, represent a novel and abundant class with pivotal regulatory functions. Over 100,000 circRNAs in human have been identified. Unlike linear mRNAs, circRNAs have no 5′ cap or 3′ poly-adenylated (poly-A) tail [6]. Instead, the 3′ and 5′ ends of circRNAs covalently link in a circle, thus forming a distinct circular structure. circRNAs are evolutionary conservative and relatively stable, and the expression of certain circRNAs are closely related with developmental stage or cell type [7, 8]. As an emerging field in this decade, circRNAs have attracted much attention due to their specific function by sponging certain microRNAs (miRNAs) or RNA-binding proteins (RBPs), and interacting with them to regulate the whole transcript profile [7–9]. Recently, deregulated expressions of certain circRNAs have been identified in many types of cancer [10–12], and are proved to play vital roles in cancer progression. All these convincing findings make circRNAs very likely to be potential prognosis biomarkers and therapeutic targets in cancer treatment. However, the role of circRNAs in CRC progression, especially in the metastasis-initiating step, has not been clearly elucidated.

In the present study, by using RNA sequencing in CRC subgroups with different metastasis-initiating ability built by Transwell assay, we identified *hsa_circ_0008558* (circLONP2) to be essential for metastasis of CRC cells, and closely associated with aggressive clinicopathological characteristics of CRC patients. Moreover, circLONP2 directly interacted with and promoted the processing of primary microRNA-17 (pri-miR-17), through recruiting DGCR8/Drosha complex in DDX1-dependent manner. Meanwhile, upregulated mature miR-17-5p could be assembled into exosomes and transferred to neighboring cells, resulting in dissemination of metastasis-initiating ability among CRC cells in primary site. These findings, collectively, suggested that upregulated circLONP2 is a critical metastasis-driver of CRC and could serve as a novel prognostic biomarker and/or potential anti-metastasis therapeutic target in clinical practice.

## Methods

### Cell culture

The CRC cell lines DLD-1, HCT116 and SW480 were cultured in Roswell Park Memorial Institute (RPMI) 1640 medium with 10% fetal bovine serum (FBS) (Gibco, NY, USA). HEK293T cell was cultured in Dulbecco’s modified Eagle’s medium (DMEM) with 10% FBS (Gibco, NY, USA). Culture conditions were carried out at 37 °C in an incubator (Thermo Fisher Scientific, Waltham, MA, USA) with 5% CO2 and 95% air.

### Patients and samples

All primary CRC tissue samples were obtained from patients with CRC during operation between January 2007 and December 2012 at the Sun Yat-Sen University Cancer Center (SYSUCC), Guangzhou, China. The inclusion criteria for selecting CRC cases were as follows: clear imaging and pathological diagnosis, complete follow-up data. And the exclusion criteria were the presence of previous treatment, both locally or systemically. Tumor stage was defined following the rules of the 2002 American Joint Committee on Cancer/ International Union Against Cancer tumor-node-metastasis (TNM) classification system. All samples were collected with informed consent of patients under institutional review board-approved protocols and stored at − 80 °C in SYSUCC Bio-bank until use.

### Transwell assay and invasion assay

Transwell assay and invasion assay were applied using 24-well insert, 8 μm pore size with or without pre-coated matrigel from Corning Inc., according to the manufacturer’s directions. In brief, 500 μL RPMI 1640 medium supplemented with 30% FBS was loaded into the lower side of the Transwell chamber, while 1 × 10^5^ cells in 250 μL FBS-free RPMI 1640 medium were loaded into the upper side. Then 24 h later, cells penetrated to the underside of the membrane were fixed and stained, and further counted in 5 random fields under a microscope.

In order to screen out CRC subgroups with different metastatic ability, we recycled cells distributed in or out of the well, respectively. After five generations of migration assay and three generations of invasion assay subsequently, HM subtype derived from DLD-1 and HCT116 (DLD-1-HM, HCT116-HM, respectively), and LM subtype derived from DLD-1 (DLD-1-LM), were built.

### RNA-sequencing and analysis of circRNAs

All the RNA-sequencing reads were first mapped to the human reference genome (GRCh37/hg19). The unmapped reads were then used to identify circRNAs using find_circ [8] and CIRI2 [13]. The raw counts were first normalized using TPM [14]. Prior to differential gene expression analysis, for each sequenced library, the read counts were adjusted by edgeR program package through one scaling normalized factor. Differential expression analysis of two samples was performed using the DEGseq (2010) R package. *P* value was adjusted using q value. Q value 0.01 was set as the threshold for significantly differential expression.

### Silencing and overexpressing of circLONP2

circLONP2-specific ASO was synthesized by RiboBio (Guangzhou, China), specifically targeting the junction sequence. Sequences for ASOs used in this article are listed in Supplemental Table S1. The overexpression plasmid of circLONP2 was constructed by ForeverGen (Guangzhou, China).

### Microarray analysis of miRNAs

DLD-1 cells were transfected with si-circLONP2 and negative control, or circLONP2-overexpressing vector and the empty control. Then, total RNA was extracted and transcribed. RNA quantity and quality were measured by NanoDrop ND-1000. RNA integrity was assessed by standard denaturing agarose gel electrophoresis. The Whole Human miRNA Microarray was a broad view that represents all known miRNAs in the human transcriptome. Sequences were compiled from a broad source survey, and then verified and optimized by alignment to the assembled human transcriptome. Double-stranded cDNA was labeled using the Quick Amp Labeling kit (Agilent Technologies, Palo Alto, CA) and hybridized to the Arraystar (Rockville, MD) Human miRNA Microarray 8 × 60 K. Following the washing steps, the arrays were scanned by the Agilent Scanner G2505C, and array images were analyzed using the Agilent Feature Extraction software (version 11.0.1.1). Quantile normalization and subsequent data processing were performed using the GeneSpring GX v12.1 software (Agilent Technologies). After quantile normalization of the raw data, miRNAs that at least 3 out of 6 samples have flags in Detected (“All Targets Value”) were chosen for further data analysis. Differentially expressed miRNAs with statistical significance between the two groups were identified through Volcano Plot filtering. Differentially expressed miRNAs between the two samples were identified through Fold Change filtering. Hierarchical Clustering was performed using the R scripts.

### RT-qPCR

Total RNA was extracted using TRIzol reagent (Invitrogen, Carlsbad, CA, USA). RNA concentration was measured by NonoDrop 2000, and each paired sample was adjusted to the same concentration. RT-qPCR was performed as described. All the results were normalized to Glyceraldehyde 3-phosphate dehydrogenase (GAPDH), β-actin, U6, λ- polyA or cel-miR-39. The primers used in this article are listed in Supplemental Table S1.

### Fish

After trypsinization, centrifugation and washed in PBS for two times, DLD-1 cells transfected with circLONP2-overexpressing vector and control empty vector were fixed in fixative liquid for 30 min. The fixative liquid was made up by methanol and acetic acid with a ratio of 3:1. Then the cell suspension was dropwise added to a clean slide, and spread evenly. After air-drying, all the slides were incubated through 70–80%-100% ethanol series at room temperature for 2 min each to dehydrate. Meanwhile, the circLONP2-specific probe was denatured at 80 °C for 10 min. Add 10–15 μL hybridization-mix, made up by RNA probe and hybridization liquid, onto each slip, then cover and seal them carefully with sealing film. Incubate all the slides in a moist chamber containing 2 × SSC and 50% methanamide at 37 °C for 12–16 h. Wash the slides by 2 × SSC for 2 times after treated by 100% ethanol for 2 min. Add 2–3 drops of HRP-conjugated streptavidin contained in Alexa Fluro 488 Tyramide SuperBoostTM Kits (Invitrogen, Carlsbad, CA, USA), and incubate for 1 h at room temperature. After rinsing the slides with 1 × PBS for 3 times, incubate them with 100 μL Tyramide Working Solution each for 2–10 min at room temperature. Then 100 μL Reaction Stop Reagent was added onto the slides. Add 100 μL 4′,6-diamidino-2-phenylindole (DAPI) (Beyotime, Shanghai, China) to each slide and incubate for 5 min to stain the nuclei. Wash all slides with 2 × SSC for 3 min. Use a confocal laser scanning microscope to analyze the subcellular location of circLONP2.

The sequence of the circLONP2-specific probe was: 5′-biotin-CAG GCA UGC UGC CAA CAU AGG UGC GCC UUC UCU CUC AGU CAC AUC AGA ACG GUC CAA CUU-3′.

### RNA pulldown

Whole-cell lysate from CRC cell lines and HEK293T cells with circLONP2 stably overexpressed were mixed with biotin-labeled circLONP2-specific probe and negative control probe, respectively. These mixtures were supplemented with tRNA (Ambion, Austin, TX, USA) to a final concentration of 0.1 μg/μL and then incubated at 4 °C overnight with gentle rotation. About 30 μL of prewashed Streptavidin magnetic beads (Invitrogen, Carlsbad, CA, USA) were added for 4–5 h at 4 °C after that. The RNA reserved in the beads was extracted by TRIzol (Invitrogen, Carlsbad, CA, USA) and further detected by RT-qPCR and normalizing to β-actin, while the protein reserved in that was detected by WB using anti-DGCR8 (10996–1-AP, Proteintech, China) rabbit antibodies. In addition, the sequence of the circLONP2-specific probe was the same as that used in FISH.

### RIP

Immunoprecipitations were performed using anti-FUS (11570–1-AP, Proteintech, China), anti-DGCR8 (10996–1-AP, Proteintech, China), anti-DDX1 (11357–1-AP, Proteintech, China) or anti-FLAG (14,793, CST, USA) antibody previously bound to magnetic Dynabeads (Life Technologies, USA) in the RIP Immunoprecipitation Buffer (Magna RIP Kit) and incubated with DNA-free fragmented RNAs. Beads were then treated with proteinase K (20 mg/mL) for 1.5 h at 65 °C. RNAs was extracted by phenol: chloroform: isoamyl alcohol and subjected to RT-qPCR using primers for pri-miRNAs and normalizing to input.

### circRIP

The circRIP assay was performed as previously described with minor modification. CircLONP2-overexpressing HEK293T cells were fixed by 1% formaldehyde for 10 min, lysed, and sonicated. After centrifugation, 50 μL of the supernatant was retained as input and the remaining part was incubated with a circLONP2-specific probes and streptavidin dynabeads (Life Technologies, USA) mixture overnight at 30 °C. On the next day, a dynabeads-probes-circRNAs mixture was washed and incubated with 200 μL of lysis buffer with or without proteinase K to reverse the formaldehyde cross-linking. Finally, the mixture was added with TRIzol (Life Technologies, USA) for RNA extraction and detection.

### Northern blot

NorthernMax™ Kit (Invitrogen, Carlsbad, CA, USA) were used according to the manufacturers’ protocols. Briefly, 10 μg total RNA mixed with an equal volume of Glyoxal Load Dye was loaded to the wells of the gel after incubating 30 min at 50 °C and then run at ~ 5 V/cm. Following the electrophoresis, RNA was transferred to a nylon membrane (GE Healthcare, Little Chalfont, Buckinghamshire, UK) overnight. After 30 min of pre-hybridization, the membrane was hybridized for 16 h at 55 °C in ΜLTRAhyb buffer containing the denatured probe. After the blocking and washing steps, the membrane was detected using the Chemiluminescent Nucleic Acid Detection Module Kit (Thermo Fisher Scientific, Waltham, MA, USA).

The sequence of the circLONP2-specific probe was the same as that used in FISH. The sequence of the GAPDH-specific probe was: 5′-UAU CCA CUU UAC CAG AGU UAA AAG CAG CCC UGG UGA CCA GGC GCC CAA UAC GAC CAA A-biotin-3′.

### Exosome experiments

For exosome extraction, cells were cultured in medium with exosome-free FBS, which was prepared by centrifugations to remove existing exosomes. Then, we followed the standard centrifugation steps. Briefly, centrifugation at 300 g for 10 min, followed by 2000 g for 20 min, and 10,000 g for 30 min. The supernatant was then filtered through a 0.2-μM filter (Pall Corp.) and further centrifuged at 100,000 g for 70 min at 4 °C twice to pellet the exosomes. Finally, exosomes were resuspended in PBS (usually 50 μl to 100 μl).

At first, samples acquired following aforementioned procedure were examined by electron microscopy to confirm the typical characteristics as exosomes. Samples were loaded into nanoscale bronze grating after negative staining by uranyl acetate, and detected by transmission electron microscopy (TEM) to analyze the diameter and shape. NanoSight NS300 instrument equipped with NTA 3.0 analytical software (both from Malvern Instruments Ltd.) was used to analyze the size and concentration of exosomes directly based on particle Brownian motion. MicroBCA Protein Assay Kit (Thermo Fisher Scientific) was used to analyze the concentration of exosomal proteins indirectly following manufacturer’s instructions.

Electroporation of miR-17-5p mimics or inhibitors into exosomes was performed using Gene Pulser Xcell™ Electroporation System (BioRad, USA) as previously described with some changes [15]. Briefly, 3 μg exosomes and 500 nmol RNA were mixed in 400 μl of electroporation buffer and electroporation was done at 350 V in a 4 mm cuvette. The mixture was incubated at 37 °C for 30 min to make the exosomal membrane fully recovered. Then the mixture was treated with RNase to remove unincorporated RNAs. Cy5-labeled RNA in exosomes were quantified by detecting the fluorescence under fluorescent microscopes.

### Co-culture system

Cells were co-cultured indirectly in a 24 transwell plates. HM or circLONP2 overexpressing cells (or cells transfected with cy5-miR-17-5p) were cultured in the inner chamber, and untreated cells were cultured in the outer chamber. 24 to 48 h later, cells in the inner chamber were taken out to examine the migration/invasion ability, or the fluorescence.

### Animal study

Athymic nude mice were purchased from Vital River Laboratories (Beijing, China), housed under standard conditions in the animal care facility at the Center of Experimental Animal of Sun Yat-Sen University. DLD-1 cells with circLONP2 stably overexpressed or the negative control cells (2 × 10^6^ cells in 0.1 ml of FBS-free culture medium) were injected to the lateral tail vein of 5–6 weeks-old male athymic nude mice (*n* = 5/group). Untreated DLD-1 cells were injected to the lateral tail vein of nude mice, and 4 weeks later, ASO-circLONP2 or ASO-NC were injected every 3 days for 4 times in total. Eight weeks (for circLONP2-overexpression and control group) or 12 weeks (for ASO-circLONP2 and control group) after the first injection, mice were sacrificed, tissue from lung was excised, and the number of tumor nodules formed in the respective organs was counted and analyzed by hematoxylin and eosin (HE) staining. All of the procedures were approved by the Sun Yat-Sen University Animal Care and Use Committee.

### Statistical analysis

Paired or unpaired student’s t-test, χ2 test or Mann-Whitney U test were used for continuous data wherever appropriate. ANOVA was used for multiple comparisons with more than two groups. Statistical significance was defined as a *P* value of less than 0.05.

## Results

### circLONP2 is highly expressed in metastasis-initiating subgroups of CRC

**Fig. 1:**
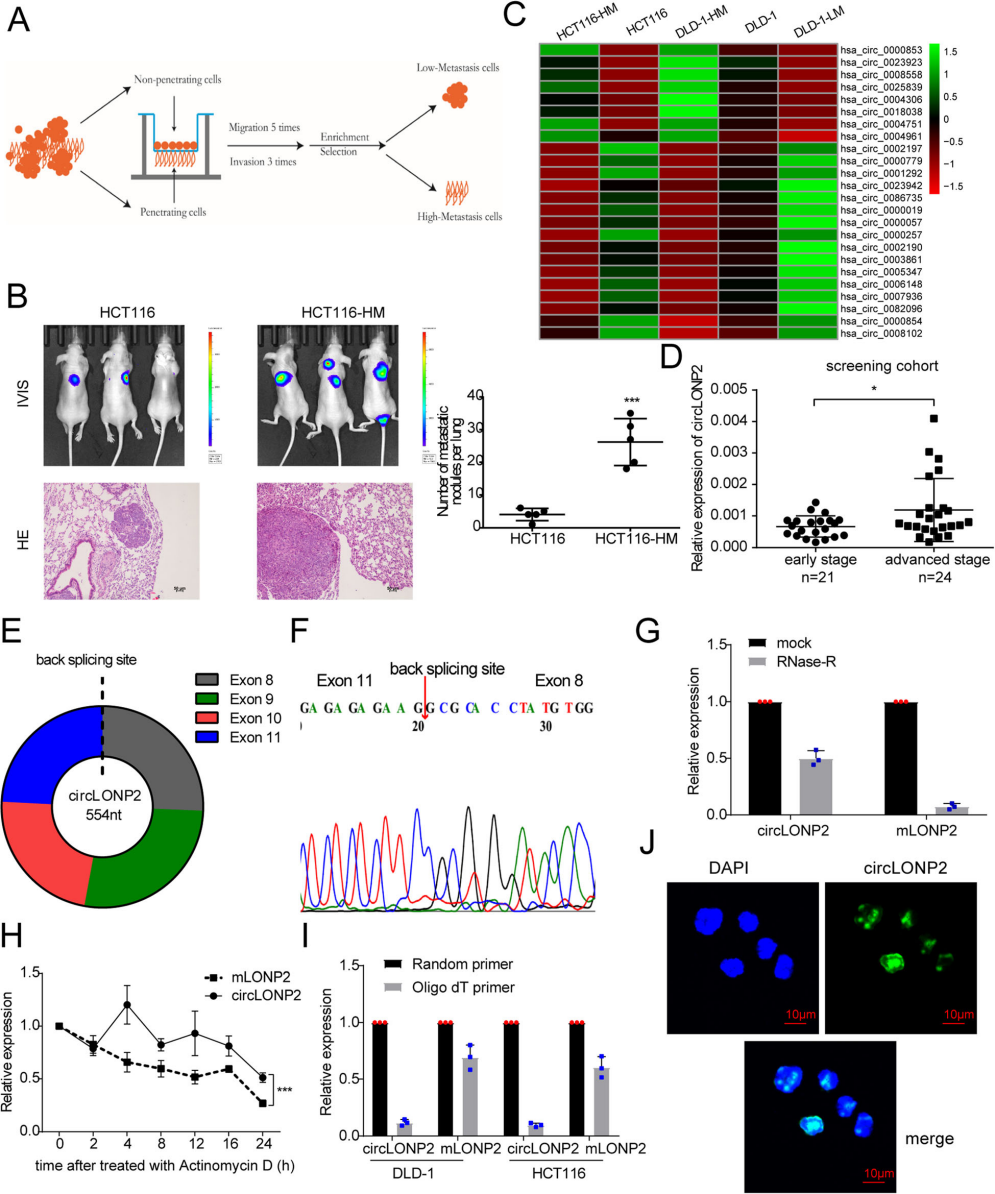
Upregulation of circLONP2 in metastasis-initiating subgroups of CRC. **a** Brief sketch map for the Transwell screening method. **b** In vivo tail vein injection model to verify the metastatic potential of different CRC subgroups by IVIS. **c** All 24 differentially expressed circRNAs in CRC subgroups exhibited by heat map. **d** Detection of circLONP2 in screening cohort by RT-qPCR (*n* = 45), normalized to GAPDH. **e** Sketch map for circLONP2. **f** Sanger sequencing to confirm the specific back splicing site of circLONP2. **g**-**i** RNase-R and actinomycin D treatment, and RT-qPCR using oligo dT primer to confirm the circular characteristics of circLONP2, normalized to GAPDH. **j** RNA FISH showing the predominant nuclear distribution of circLONP2 in HCT116. IVIS, in vivo imaging system. All experiments were repeated for at least three times, data were shown as mean ± SD, * *P* < 0.05, ** *P* < 0.01, *** *P* < 0.001 in independent Student’s t test (**b**), Mann-Whitney U test (**d**), or two-way ANOVA (**h**)

To investigate potential CRC subgroups and key driver molecule responsible for metastasis-initiation, we applied screening assay to mimic invasion step in vitro as previously described [16, 17] (Fig. 1a), and built CRC subgroups with different metastatic potential (high metastatic potential cells, HM; low metastatic potential cells, LM; normal parental CRC cells, N). Then the invasive ability and other characteristics of these subgroups were reassessed and proved by in vitro and in vivo assays (Supplemental Fig. S1A-E, Fig. 1b). Subsequently, high throughput RNA sequencing was carried out to identify deregulated circRNAs engaged in this metastasis-initiating step (Supplemental Fig. S1F, Fig. 1c). Meanwhile, by using real-time quantitative polymerase chain reaction (RT-qPCR), we identified *hsa_circ_0008558* to be the only one upregulated in both metastasis-initiating CRC subgroups and CRC tissues with established liver and/or lymph node metastasis (screening cohort, *n* = 45) (Supplemental Fig. S1G, Fig. 1d).

According to circBase [18], *hsa_circ_0008558* was consisted of LONP2 exons 8–11, referred to as circLONP2 hereafter (Fig. 1e). Then we performed a series of experiments to confirm the existence and circular characteristic of circLONP2. Sanger sequencing examined the specific back-splicing sequence (Fig. 1f), and RNase-R or actinomycin D treatment assay (Fig. 1g, h) showed that circLONP2 was relatively resistant to RNase-R and more stable than the linear messenger RNA of LONP2 (mLONP2). Moreover, Fig. 1i indicated that circLONP2 had no poly-A tail. And RNA fluorescence in situ hybridization (FISH) showed the predominant nuclear distribution of circLONP2 in HCT116 cell (Fig. 1j). Additionally, we proved that circLONP2 could not be translated by using an overexpression plasmid of circLONP2 fused with eGFP (Supplemental Fig. S1H-J).

### circLONP2 correlates with metastasis and unfavorable prognosis of CRC patients

**Fig. 2:**
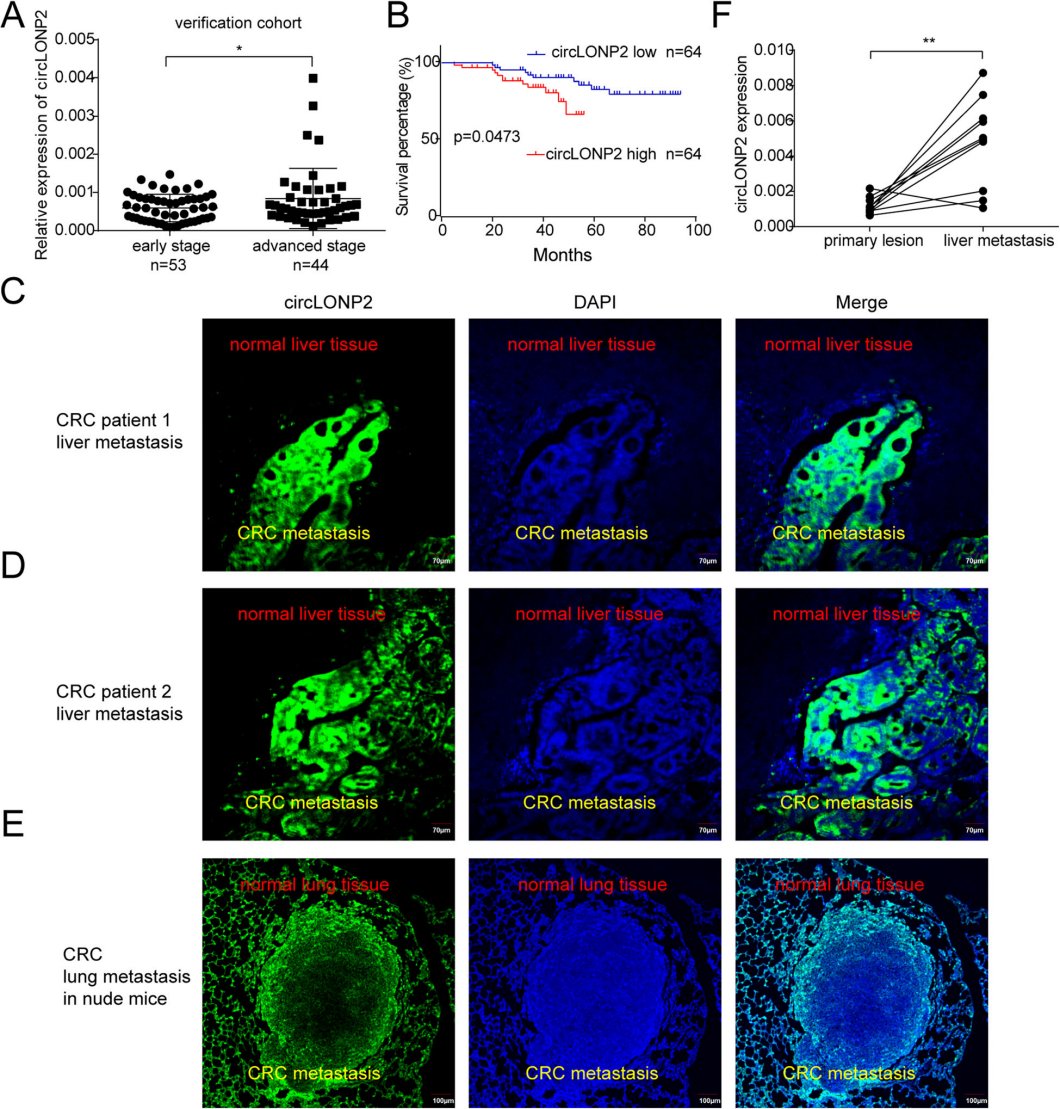
circLONP2 correlates with metastasis and unfavorable prognosis of CRC patients. **a** Detection of circLONP2 in verification cohort by RT-qPCR, normalized to GAPDH (*n* = 97). **b** Kaplan-Meier survival analysis of CRC patients according to the expression level of circLONP2 using log-rank test (*n* = 128). **c**-**e** RNA FISH in tissue specimen showing significant circLONP2 overexpression along with the invasive margin of metastatic CRC. **f** Detection of circLONP2 in primary CRC tissue and paired liver metastasis, normalized to GAPDH (*n* = 10). All experiments were repeated for three times, data were shown as mean ± SD, * *P* < 0.05, ** *P* < 0.01 in Mann-Whitney U test (**a**), log-rank test (**b**), or paired Student’s test (**f**)

Next, we examined the expression of circLONP2 in another verification cohort consisted of 97 primary CRC tissues, and found that circLONP2 was consistently and significantly increased in CRC tissues with advanced-stage (Fig. 2a, *n* = 97). Furthermore, Kaplan-Meier analysis showed that CRC patients with increased expression of circLONP2 had shorter overall survival (Fig. 2b, *n* = 128, all primary CRC tissues used in screening and verification cohort, except for 14 tissues with incomplete clinical data), and circLONP2 was proved to be an independent predictor by using Cox regression analysis (Supplemental Table S2). Meanwhile, correlation analysis demonstrated that high expression of circLONP2 was positively correlated with aggressive clinicopathological characteristics, especially tumor metastasis and advanced clinical stage (Supplemental Table S3). According to previous studies, metastasis-related oncogenes were frequently overexpressed in metastatic site, especially along the edge of tumor [3]. To further confirm its correlation with metastasis, by using RNA FISH, we found that the expression pattern of circLONP2 was always in correspondence with the penetrating tendency of CRC cells in both liver and lung metastases (Fig. 2c-e). Additionally, the expression of circLONP2 was significantly higher in liver metastasis than that in paired primary CRC tissue (Fig. 2f, *n* = 10). All these findings suggested that circLONP2 may play vital role in CRC metastasis.

### The biogenesis of circLONP2 is modulated by FUS

**Fig. 3:**
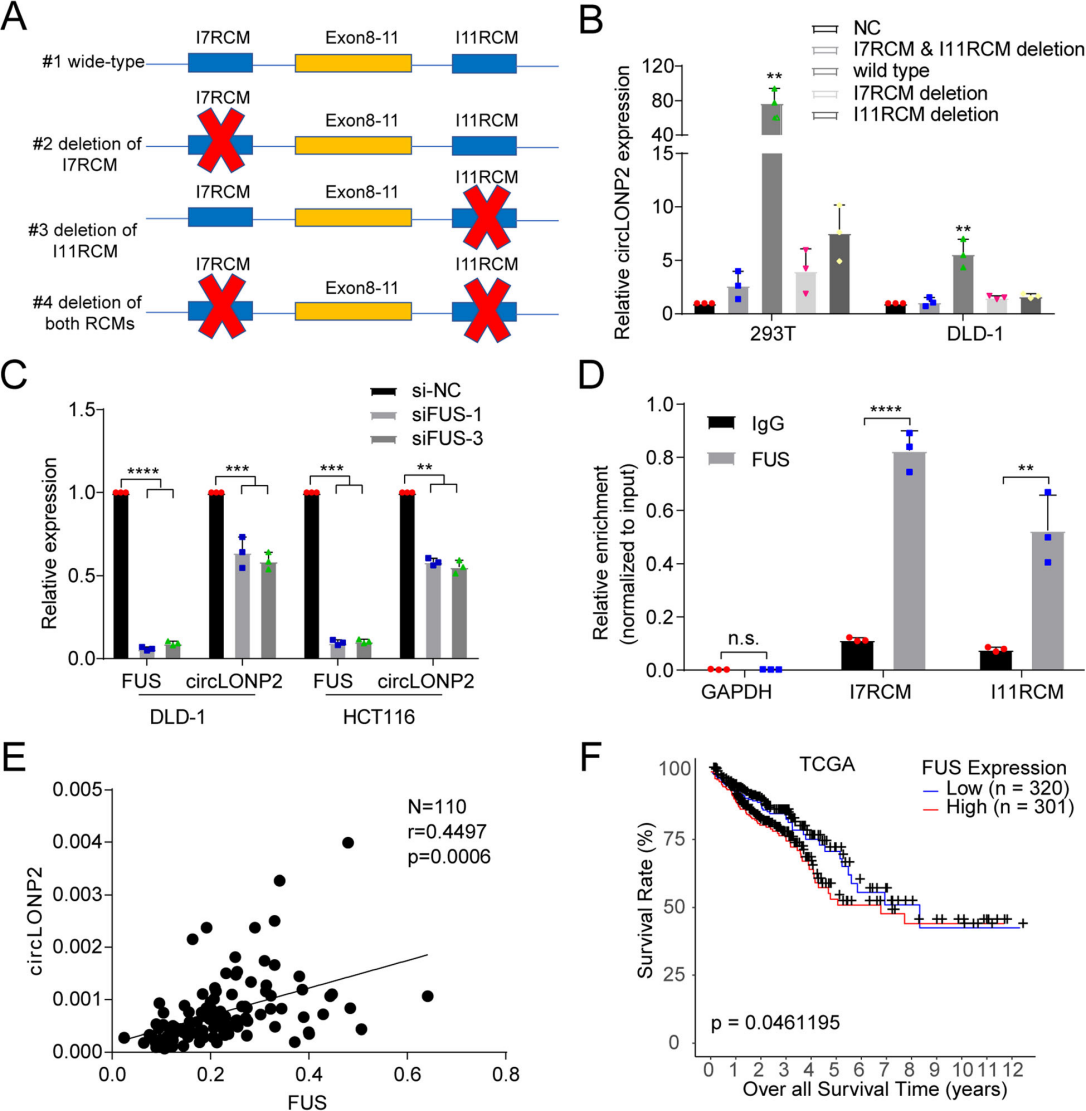
The biogenesis of circLONP2 is regulated by FUS. **a** Different deletion mutants for circLONP2-overexpressing plasmid. **b** RT-qPCR showed that only the wild type could significantly and stably overexpress circLONP2, normalized to GAPDH. **c** Knockdown of FUS significantly decreased the expression of circLONP2, normalized to GAPDH. **d** anti-FUS RIP showed significant enrichment of both I7RCM and I11RCM. **e** The expression of FUS and circLONP2 in CRC tissue showed significant positive correlation, normalized to GAPDH. **f** The mRNA level of FUS were correlated with prognosis of CRC patients in TCGA database. All experiments were repeated for three times, data were shown as mean ± SD, * *P* < 0.05, ** *P* < 0.01, *** *P* < 0.001, **** *P* < 0.0001 in one-way ANOVA (**b**, **c**), Mann-Whitney U test (**d**), person correlation test (**e**) or log-rank test (**f**)

It was previously reported that reverse complementary matches (RCMs) are a conserved feature of circRNA biogenesis [19]. Therefore, we aligned the sequence of introns flanking circLONP2 to find out all possible RCMs. As predicted, two highly matched RCMs (84% identity over 129 nucleotides, Supplemental Fig. S2A) were observed, and termed I7RCM (RCM in intron 7) and I11RCM (RCM in intron 11), respectively. We cloned and connected them with linear sequences of exon 8 to exon 11 to a pcDNA3.1 vector (#1 wide-type), and a series of deletion constructs (#2-#4, deletion of I7RCM, deletion of I11RCM and deletion of both RCMs, respectively) (Fig. 3a). After transient transfection, RT-qPCR showed that the wide-type vector (#1), but not the other mutants (#2-#4), could significantly overexpress circLONP2 (Fig. 3b), indicating that I7RCM and I11RCM are responsible for biogenesis of circLONP2. Northern blot assay further confirmed this result (Supplemental Fig. S2B).

Since Circular RNA Interactome [20] predicted that the well-known proto-oncogene, FUS, may bind with the flanking regions of circLONP2, and it was documented that FUS could regulate circRNA expression in murine embryonic stem cell-derived motor neurons [21], we then investigated if the expression of circLONP2 was regulated by FUS. As anticipated, circLONP2 was downregulated upon knocking down FUS by small interfering RNA (siRNA) (Fig. 3c, Supplemental Fig. S2C). Furthermore, I7RCM was consisted of several repeated “GUUG” or “ACUU” regions, which were coincidently among the binding motifs of FUS (Supplemental Fig. S2D) [22]. In fact, anti-FUS RNA immunoprecipitation (RIP) did reveal a significant enrichment of I7RCM and I11RCM (Fig. 3d). Moreover, the expression of circLONP2 was positively correlated to the mRNA level of FUS in primary CRC tumor tissues (Fig. 3e, *n* = 110), and FUS mRNA expression was significantly correlated with clinicopathological characteristics (Supplemental Table S4). Additionally, we found that the expression of FUS mRNA was negatively correlated with the overall survival rate of CRC patients from TCGA database (Fig. 3f). Meanwhile, the expression of FUS protein in CRC tumor tissue was significantly increased than that in paired para-tumor tissues (Supplemental Fig. S2E).

### circLONP2 is essential for CRC metastasis

**Fig. 4:**
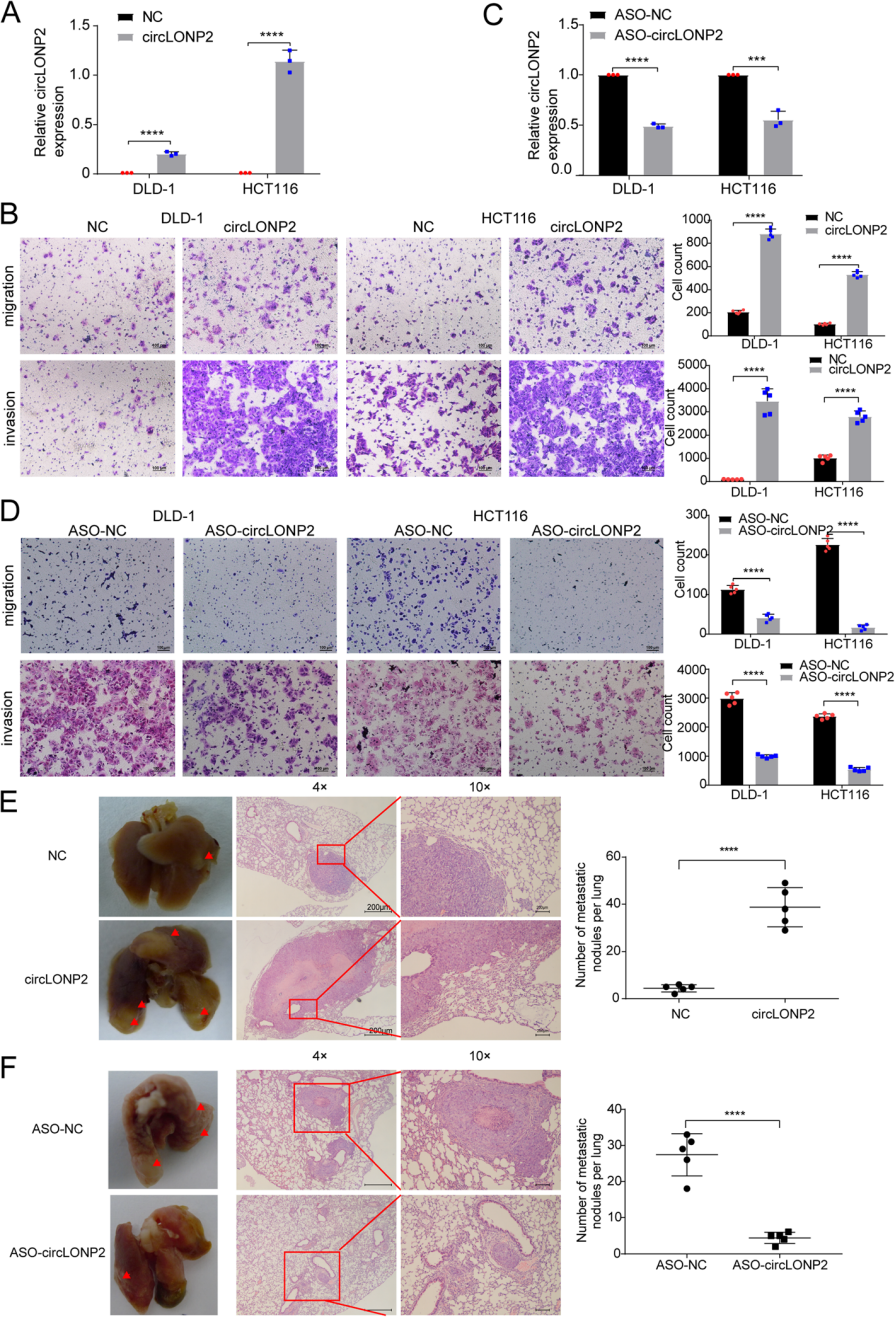
circLONP2 is essential for CRC metastasis**. a**, **b** Overexpression of circLONP2 significantly enhanced the migration and invasion ability of CRC cells. **c**, **d** Knockdown of circLONP2 by ASO significantly suppressed the migration and invasion ability of CRC cells. **e**, **f** In vivo tail vein injection model confirmed that overexpression or knockdown of circLONP2 could significantly promote or attenuate CRC cells metastasize to lung, respectively. All detection of circLONP2 by RT-qPCR was normalized to GAPDH. All experiments were repeated for three times, data were shown as mean ± SD, * *P* < 0.05, ** *P* < 0.01, *** *P* < 0.001, **** *P* < 0.0001 in Mann-Whitney U test (**a**, **c**, **e**, **f**), or independent Student’s t test (**b**, **d**)

We next investigated the correlation between circLONP2 and metastatic ability of CRC cells. Normal parental CRC cells with enforced expression of circLONP2 demonstrated significant increase of migration and invasion abilities (Fig. 4a, b). On the other hand, by using anti-sense oligonucleotide (ASO) targeting circLONP2-specific back splicing sequence, we successfully knocked down the expression of circLONP2 (Fig. 4c), and subsequent Transwell migration and invasion assay showed significantly diminished amounts of penetrated CRC cells (Fig. 4d). In vivo tail vein injection model confirmed that ectopic overexpression of circLONP2 markedly promoted the potential of CRC cells to metastasize to lungs (Fig. 4e). And most importantly, ASO-mediated depletion of circLONP2 dramatically reduced the penetrance, including both size and numbers, of metastasis to lungs (Fig. 4f). All the results indicated that circLONP2 was essential for metastasis of CRC cells.

### circLONP2 promotes CRC aggressiveness through directly interacting with DDX1

**Fig. 5:**
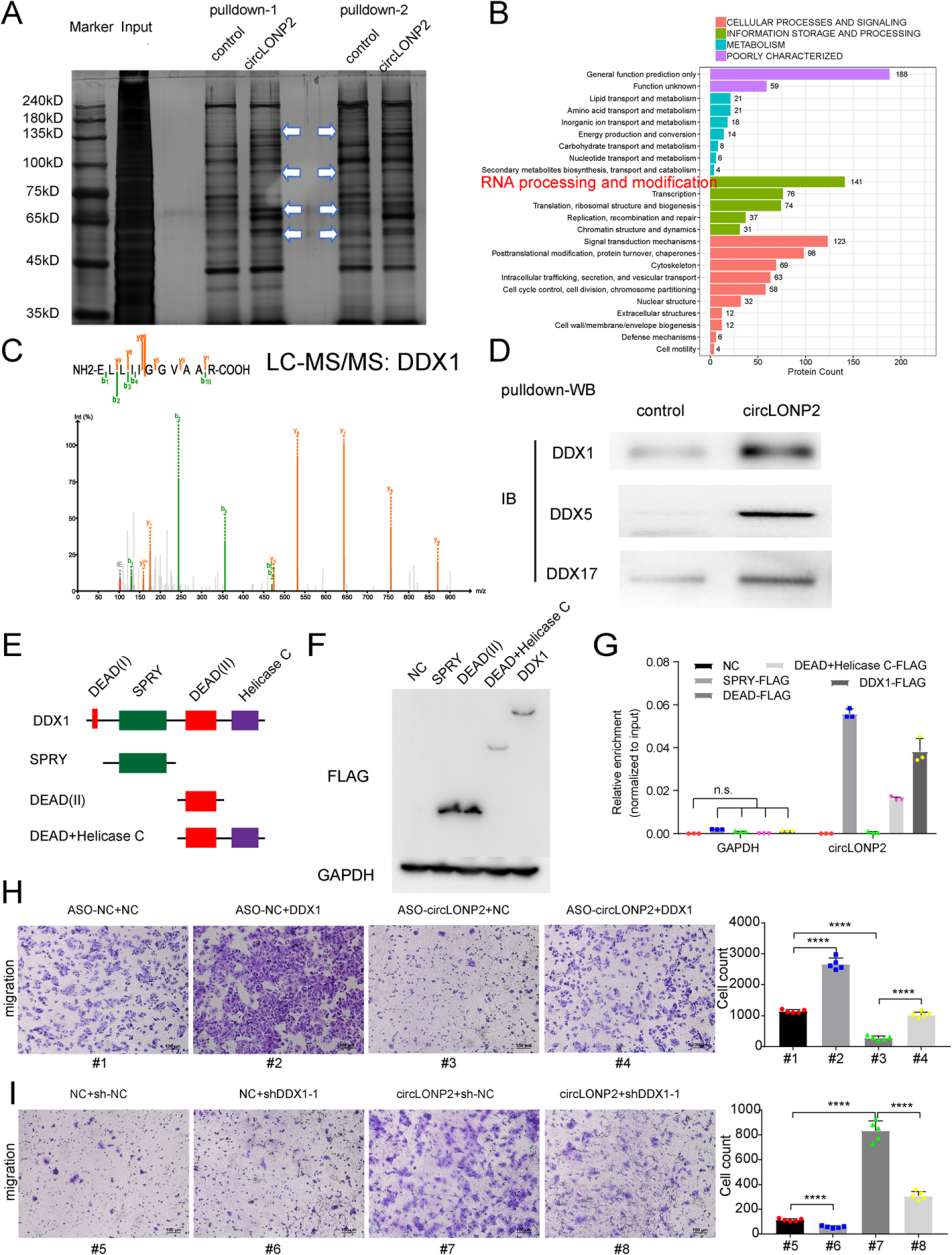
circLONP2 directly interacts with DDX1. **a** Different protein bands detected by silver stain for mass spectrometry. **b** KEGG analysis of potential proteins interacting with circLONP2. **c** The specific amino acid sequence of DDX1 showed by second-order mass spectrum. **d** RNA pulldown followed by WB confirmed the interaction of circLONP2 with DDX1, DDX5 and DDX17. **e**, **f** A series of deletion mutants of DDX1 labeled with FLAG and confirmed by WB. **g** Anti-FLAG RIP assay showed significant enrichment of circLONP2 in SPRY domain and the full length of DDX1. **h**, **i** Rescue experiments indicated that DDX1 was essential for circLONP2-enhanced migrating ability of CRC cells. All experiments were repeated for three times, data were shown as mean ± SD, * *P* < 0.05, ** *P* < 0.01, *** *P* < 0.001, **** *P* < 0.0001 in one-way ANOVA (**g**-**i**)

Studies have shown that nucleus-retained circRNAs usually perform their functions in regulating transcription and splicing through interacting with proteins [23–25]. Then we decided to investigate the protein interactome and relevant pathways in order to dissect the underlying mechanisms of circLONP2-driven metastasis-initiation of CRC in detail. After pulldown assay and silver staining, we chose differential band for mass spectrum (Fig. 5a), and enrichment analysis was carried out to find out relevant pathways that circLONP2-interacted proteins were significantly enriched in (Fig. 5b). Within the most enriched pathway, RNA processing and modification pathway, DEAD-Box (DDX) protein family, including DDX1, DDX5 and DDX17 (Fig. 5c, Supplemental Fig. S3A-B), known as vital regulatory components of the miRNA-processing machinery, attracted our attention [26, 27]. Next, we applied RNA pulldown followed by Western Blot (WB) assay, and confirmed that circLONP2 did interact with DDX family members (Fig. 5d). The oncogenic role of DDX5 and DDX17 in CRC progression has been well-established, however, the role of DDX1 in CRC progression is not yet clear. It might be more interesting to investigate whether and how DDX1 mediates the pro-metastasis role of circLONP2 in CRC [28–30].

Subsequently, we tried to investigate the exact domain of DDX1 that directly interact with circLONP2. We constructed the wild type and a series of truncated mutants of DDX1 labeled by FLAG (Fig. 5e), verified by WB assay (Fig. 5f), and transfected them together with circLONP2-overexpressing plasmid to 293 T cells. Forty-eight hours later, cells were harvested and anti-FLAG RIP assay showed that the SPRY domain was most important for the interaction of DDX1 and circLONP2 (Fig. 5g). In order to clarify the exact role of DDX1 in circLONP2-initiated CRC metastasis, we did several rescue experiments, and found that DDX1 was essential for circLONP2-enhanced migration and invasion ability of CRC cells (Supplemental Fig. S3C-F, Fig. 5h, i).

### rcLONP2 and DDX1 collaboratively modulate pri-miR-17 processing

**Fig. 6:**
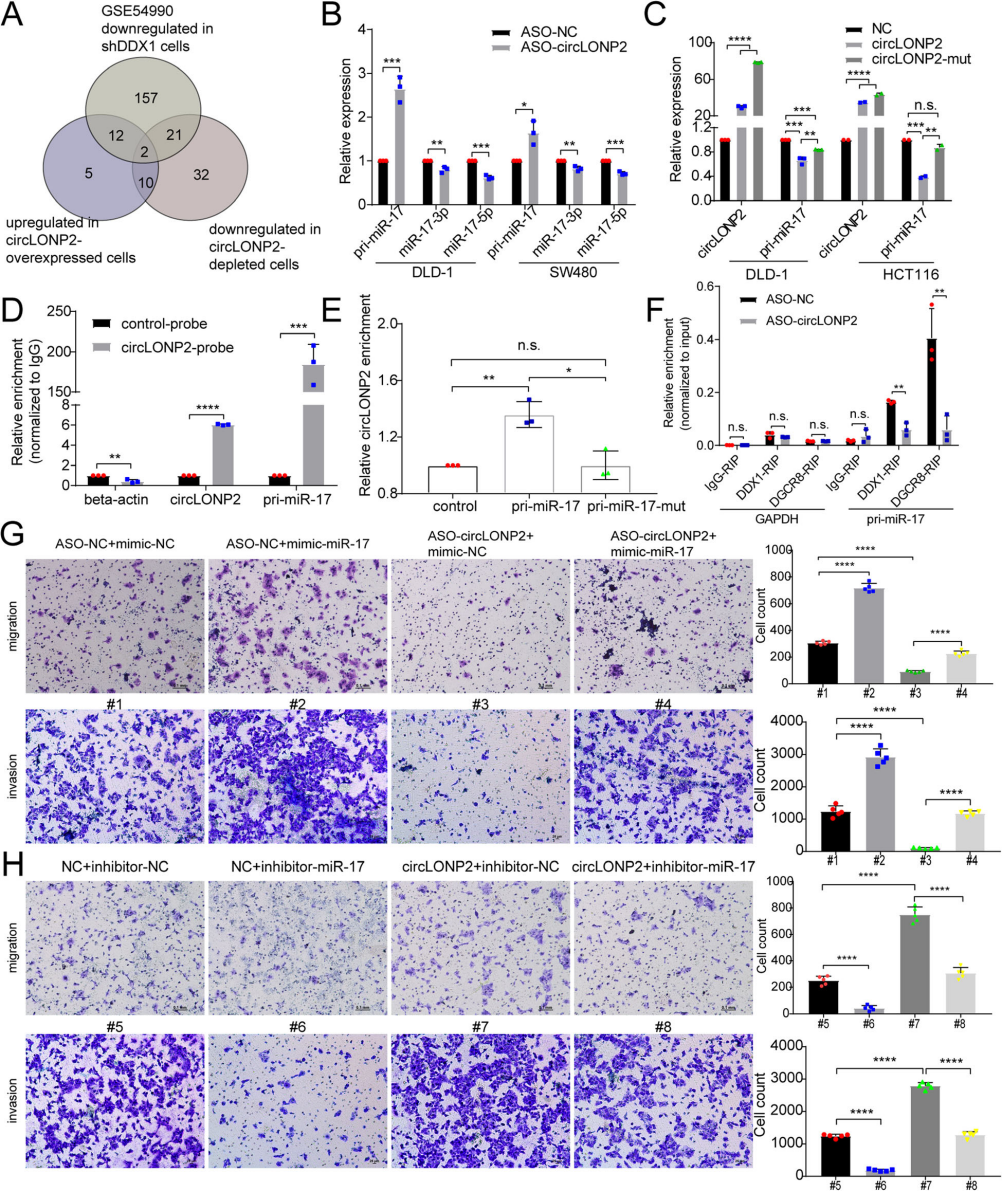
circLONP2 and DDX1 collaboratively modulate pri-miR-17 processing. **a** Venn diagram showed potential common downstream miRNA targets of circLONP2 and DDX1 according to miRNA array results. **b**, **c** Verification of pri-miR-17 as a downstream target of circLONP2 by RT-qPCR, normalized to GAPDH. **d**, **e** circRIP and affinity pulldown revealed direct interaction of circLONP2 and pri-miR-17 through specific reverse complementary sequences, respectively, normalized to GAPDH. **f** RIP followed by RT-qPCR indicated circLONP2 mediated the interaction between pri-miR-17 and DDX1 or DGCR8. **g**, **h** Rescue experiments indicated that miR-17 was essential for circLONP2-enhanced migrating ability of CRC cells. All experiments were repeated for three times, data were shown as mean ± SD, * P < 0.05, ** *P* < 0.01, *** *P* < 0.001, **** *P* < 0.0001 in Mann-Whitney U test (**b**), one-way ANOVA (**c**-**e**, **g**-**h**), or independent Student’s t test (**f**)

Due to the fact that circLONP2 has synergistic effect with DDX1 in promoting CRC migration, and DDX1 could regulate maturation of specific miRNA subgroups, we then speculated that circLONP2 may participate in miRNAs processing. To validate this hypothesis and screen for potential downstream targets, miRNA array in cells with circLONP2 depletion or overexpression were applied, and we found that circLONP2 did alter the expression patter of certain miRNAs (Supplemental Table S5). Next, all the significantly changed miRNAs were overlaid with that from GSE54990 miRNA array data set, which was carried out in cells with DDX1 knockdown (Fig. 6a). miR-17, known as an onco-miRNA in cancer development, was identified to be one of the potential common targets of circLONP2 and DDX1 (Fig. 6a, Supplemental Table S5). To further confirm the microarray findings using an independent approach and to find out whether circLONP2/DDX1-mediated deregulation of miRNAs is through modulating the processing of primary miRNAs, RT-qPCR was performed. The result revealed that mature miR-17-3p/5p were downregulated, which was in consistent with microarray findings (Fig. 6b, Supplemental Fig. S4A). Meanwhile, we detected the expression of pri-miR-17 using specific PCR primers whose efficiency were verified by DNase I treatment (Supplemental Fig. S4B), and found that pri-miR-17 was upregulated in both circLONP2 or DDX1 depleted cells (Fig. 6b). Furthermore, after treated with actinomycin D, circLONP2-knockdown could significantly increase the stability of pri-miR-17, while circLONP2-overexpression could cause the opposite effect (Supplemental Fig. S4C-D).

We further explored the underlying mechanism through which circLONP2 and DDX1 modulate the processing of pri-miR-17. It has been well established that ncRNAs may interact with specific target RNA through pairing of highly complementary bases [8, 31–33]. Then we compared the RNA sequences of circLONP2 and pri-miR-17 by using Basic Local Alignment Search Tool (BLAST) (http://blast.ncbi.nlm.nih.gov/) and identified two highly complementary regions (Supplemental Fig. S4E). In subsequent studies, we mutated the two potential binding sites on circLONP2 and found that wild-type circLONP2 could promote the processing of pri-miR-17, while mutated circLONP2 had little effect on this event (Fig. 6c). To validate the direct association between circLONP2 and pri-miR-17, we performed circRNAs in vivo precipitation (circRIP) assay using biotin-labeled circLONP2-specific RNA probe [11, 34]. The results showed that pri-miR-17 was significantly enriched in circLONP2-probe group compared with that in control-probe group (Fig. 6d). This specific interaction between circLONP2 and pri-miR-17 was further confirmed by affinity pulldown of endogenous circLONP2 using in vitro-transcribed biotin-labeled pri-miR-17 and pri-miR-17-mut (Fig. 6e).

### circLONP2 recruits DGCR8 to pri-miR-17 in DDX1-dependent manner

The maturation of miRNAs relies heavily on efficient work of the core microprocessor complex, namely, the intranuclear RNase III enzyme Drosha and the double-stranded RNA-binding protein DGCR8 [35–38]. Therefore, we speculated that circLONP2/DDX1 complex may facilitate DGCR8/Drosha binding to pri-miR-17, thus promoting its processing. To confirm this speculation, we applied endogenous co-immunoprecipitation and found that DDX1 and DGCR8 interacted with each other in RNA-independent way (Supplemental Fig. S4F). Moreover, RNA pulldown assay revealed that knocking down DDX1 significantly decreased the amount of DGCR8 that bound to circLONP2 (Supplemental Fig. S4G). Meanwhile, circLONP2-depletion dramatically attenuated the interaction between pri-miR-17 and DDX1 or DGCR8 (Fig. 6f), and overexpressing DDX1 or DGCR8 could partially rescue the accumulation of pri-miR-17 caused by circLONP2-knockdown (Supplemental Fig. S4H). All these results indicated that circLONP2 could directly bind to pir-miR-17, and indirectly recruit DGCR8/Drosha microprocessor through DDX1, to facilitate the maturation processing of pri-miR-17.

We then investigated whether circLONP2-mediated maturation of miR-17 was responsible for the increased migration and invasion ability of CRC cells. We successfully overexpressed miR-17 through specific miR-mimics (Supplemental Fig. S4I), and rescue experiments showed that overexpressing miR-17 restored the diminished migration and invasion ability of CRC cells caused by circLONP2-depletion (Fig. 6g), while inhibiting miR-17 showed opposite effects (Fig. 6h). Furthermore, the expression of miR-17-5p was significantly increased in primary CRC with advanced stage, and positively correlated with that of circLONP2 and the overall survival rate of CRC (Supplemental Fig. S4J-L). And miR-17-5p level in liver metastasis was higher than that in paired primary CRC tissue (Supplemental Fig. S4M). All these results confirmed the oncogenic role of miR-17-5p in mediating circLONP2-driven CRC metastasis.

### circLONP2-driven intercellular transfer of miR-17-5p by exosomes disseminates high metastatic potential

**Fig. 7:**
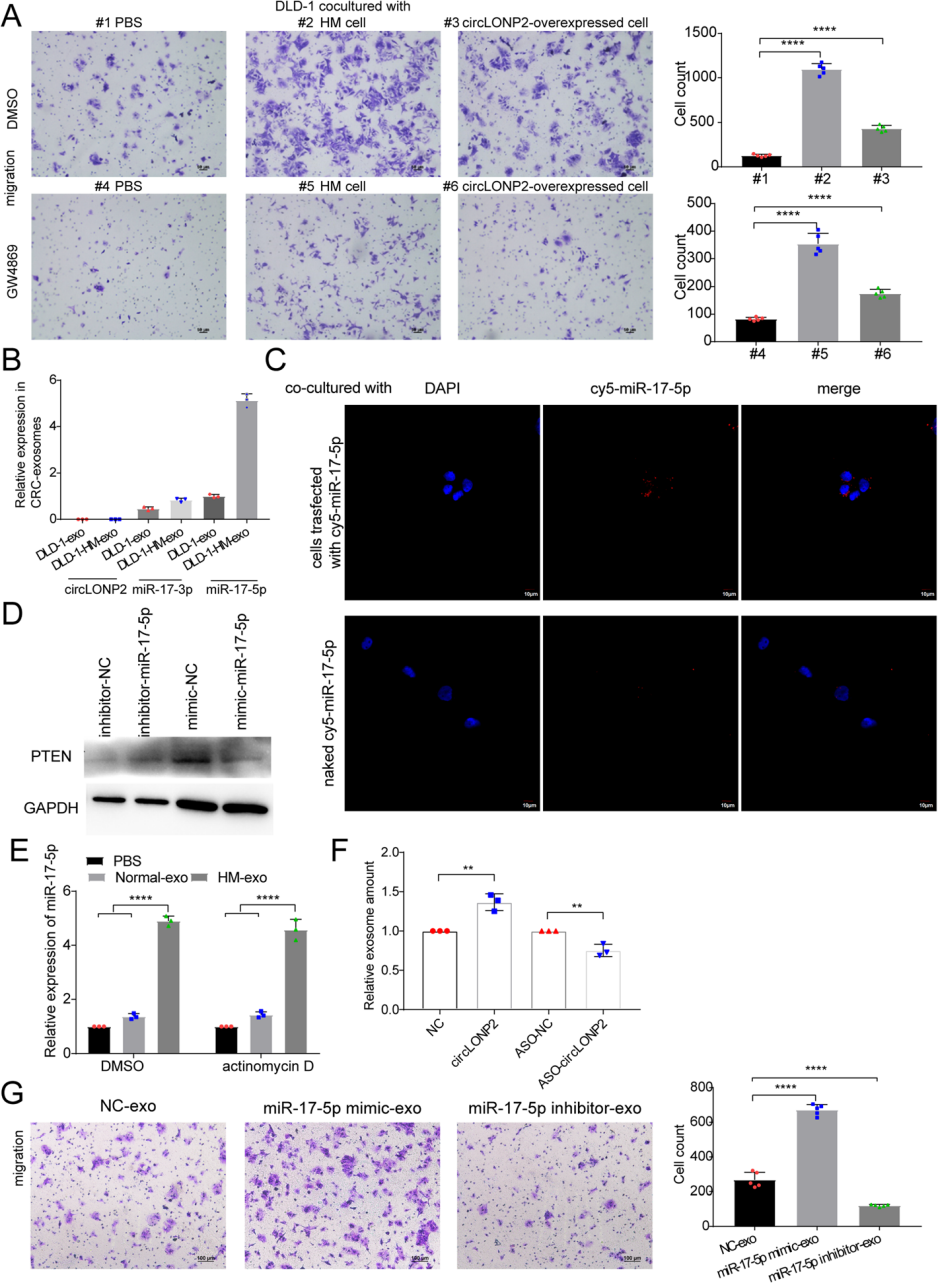
circLONP2-driven intercellular transfer of miR-17-5p by exosomes disseminates high metastatic potential. **a** DLD-1 cells co-cultured with HM or circLONP2-overexpressed cells showed enhanced migration ability, and this effect could be attenuated by GW4869 treatment. **b** Detecting the expression level of circLONP2, miR-17-3p and miR-17-5p in CRC-derived exosomes by RT-qPCR, normalized to λ-polyA or cel-miR-39. **c** Red fluorescence could be observed in almost every recipient cell co-cultured with cells transfected with cy5-miR-17-5p, whereas few fluorescence could be observed in recipient cells co-incubated with naked cy5-miR-17-5p, revealed by IF. **d** The expression of PTEN, one of classical downstream target of miR-17-5p, was significantly changed in recipient cells co-incubated with exosomes electroporated with miR-17-5p inhibitors or mimics. **e** Increase of miR-17-5p level in recipient cells after incubation with HM-exosomes could not be affected by actinomycin D treatment, normalized to U6. **f** The exosome amounts could be significantly changed upon overexpression or depletion of circLONP2, revealed by microBCA analysis. **g** CRC cells provided with exosomes containing miR-17-5p mimics exhibited enhanced metastatic ability, while exosomes containing miR-17-5p inhibitors showed opposite effect. All experiments were repeated for three times, data were shown as mean ± SD, * *P* < 0.05, ** *P* < 0.01, *** *P* < 0.001, **** *P* < 0.0001 in one-way ANOVA (**a**, **e**-**g**), or Mann-Whitney U test (**f**)

It has been well established that cancer cells with high metastatic potential could communicate with neighboring cells through the important cell-to-cell mediator, exosomes, to make the primary microenvironment more easier for metastasis-initiation [39]. As expected, parental CRC cells co-cultured with HM cells or circLONP2-overexpressed cells demonstrated significantly enhanced migration and invasion ability (Fig. 7a upper panel, Supplemental Fig. S5A upper panel), whereas suppressing the secretion of exosomes by GW4869 [40, 41] dramatically reversed this effect (Fig. 7a lower panel, Supplemental Fig. S5A lower panel). These results suggested that both circLONP2 and exosomes played critical roles in this effect.

Cell-secreted exosomes, together with their capped cargoes, such as miRNAs, lncRNAs, circRNAs and proteins, can be internalized by neighboring cells to modulate particular biological processes [42, 43]. Then we speculated that circLONP2 or its downstream targets may contribute to this process through directly being assembled into exosomes and transferred to neighboring cells. We extracted the exosomes from above-mentioned cells, and confirmed their identity by electron microscopy, WB and NanoSight analysis as previously described [32] (Supplemental Fig. S5B-D). Next, by using RT-qPCR, we found that miR-17-5p was significantly enriched in exosomes, whereas, the level of miR-17-3p was much lower, and that of circLONP2 was undetectable (Fig. 7b). To further confirm that miR-17-5p could be transferred to recipient cells via exosomes, CRC cells transfected with cy5-labeled miR-17-5p were cultured in the inner chamber of co-culture system, and red fluorescence could be observed in almost every recipient cells cultured in the outer chamber, whereas few fluorescence could be found in recipient cells co-incubated with naked cy5-miR-17-5p (Fig. 7c). Meanwhile, the expression of PTEN, one of classical downstream targets of miR-17-5p, was significantly changed in the recipient cells co-incubated with exosomes electroporated with mimic/inhibitor-miR-17-5p (Fig. 7d). Moreover, the expression level of miR-17-5p in recipient cells in recipient cells was significantly increased after incubation with HM-exosomes, and not affected by actinomycin D (Fig. 7e), which definitely excluded the possible involvement of endogenous induction. To validate whether circLONP2 was implicated in exosomal assembly of miR-17-5p, exosomes secreted from circLONP2-overexpressed or depleted cells were extracted. We found that overexpression of circLONP2 increased the amounts of exosomes and the level of miR-17-5p in exosomes, while depletion of circLONP2 showed opposite effect (Fig. 7d, Supplemental Fig. S5E). However, the expression of RAB27A and RAB27B, known as proteins vital for exosome secretion, were not changed after overexpression or depletion of circLONP2 (Supplemental Fig. S5F), indicating uncharacterized complicated regulating network in circLONP2-controled secretion of exosomes.

We further examined whether exosome-transferred miR-17-5p could confer the high metastatic potential to recipient CRC cells. CRC cells provided with exosomes that were electroporated with miR-17-5p mimics also exhibited enhanced migration and invasion ability, while exosomes electroporated with miR-17-5p inhibitors showed opposite effect (Fig. 7g, Supplemental Fig. S5G). In addition, we noticed that the exosome-conferred high metastatic potential in recipient cells could sustain for at least 5 days after the removal of exosomes extracted from circLONP2-overexpressed cells (Supplemental Fig. S5H). These data indicated that circLONP2-driven exosomal package and transfer of miR-17-5p induced sustainable high metastatic potential in recipient cells.

## Discussion

Metastasis causes the vast majority of cancer-related death [1]. The 5-year survival rate ranges from over 90% in stage I patients to merely 10% in stage IV patients of CRC [44]. Despite all the therapeutic advances in past decades, few drugs, if not none, could explicitly prevent occurrence of macroscopic metastases in foreign organs. The harsh reality that little is known about the underlying mechanisms whereby specific subgroup of cancer cells detach from the primary lesion to initiate metastasis, greatly hampers the development of metastasis-preventive therapies. In the present study, we simulated the initial phase of metastasis in vitro as previously described [16, 17], and identified metastasis-initiating subgroups in primary CRC. The key characteristics of these subgroups, i.e. significantly enhanced metastatic potential with no influence on EMT markers, were consistent with characteristics of metastasis-initiating cells in literature, indicating that EMT might not be required for the metastatic process of cancer [45–47]. circLONP2 was upregulated in metastasis-initiating subgroups and in primary CRC lesion with established metastasis, and significantly overexpressed along the invasive edge in metastatic site. Most importantly, the use of ASO to suppress circLONP2 caused pronounced inhibition of metastasis in immunodeficient mouse models of CRC. Clinically, the presence of circLONP2-overexpressed metastasis-initiating cells correlated with unfavorable prognosis of CRC patients. All these findings hinted a metastasis-initiating role of circLONP2 in CRC development and were consistent with the key traits of metastasis-related oncogenes in literature [3].

It has been well demonstrated that a large proportion of circRNAs are located in the cytoplasm and the most crucial mechanism through which they perform regulatory functions is miRNA sponge [7–9, 48]. However, circRNAs with predominant nuclear localization are extremely rare [24], and the possible molecular functions and underlying mechanisms of these circRNAs remain largely undefined. Herein, we identified circLONP2 to be distributed mainly in the nucleus and serve as a novel cofactor in microprocessor complex. Its regulatory potential in initiating metastasis of CRC cells depended on interacting with DDX1 and modulating pri-miR-17 processing.

As we all know, aberrant miRNA expression is closely related to tumorigenesis and development of human cancers [49–52]. Due to the suppression of Drosha and Dicer, the global level of miRNAs in cancer is downregulated [53]. However, miRNome studies using cancer cells have found many miRNAs to be upregulated, which apparently can’t be ascribed to the disorder of microprocessor complex [54]. Accumulating evidence has shown that post-transcriptional regulation, rather than transcriptional regulation, is critical in determining the levels of mature miRNAs [26]. Subsequent studies have identified several RBPs, such as DDX1, DDX5, DDX17, TDP-43, KSRP and SMAD4, to be regulatory components that interact with miRNA processing machinery and guide the maturation of specific subsets of miRNAs [26, 36, 38, 55, 56]. In particular, some studies showed that ncRNA-ncRNA interaction through complementarity of specific nucleotides could control pri-miRNA processing [57]. Our present work showed that circLONP2 could directly interact with and promote the processing of pri-miR-17, through recruiting DGCR8/Drosha complex in DDX1-dependent manner. This is the first time to identify a circRNA as accessory component of microprocessor, which would expand the research on potential regulatory functions of circRNAs in both physiological and pathological processes.

Oncogenic molecules could be transferred from metastasis-initiating tumor cells to their microenvironment, including but not limited to neighboring tumor cells, stromal cells, immune cells and even non-cancerous cells, through extracellular vesicles or exosomes [39, 58]. Particularly, the potential role of miRNAs packaged within exosomes in promoting cancer progression has been emphasized a lot [58–60]. In this study, we found that circLONP2-driven exosomal-assembled miR-17-5p derived from metastasis-initiating cells could be internalized by neighboring CRC cells and confer high metastatic potential. This intercellular transfer of miR-17-5p could accelerate the invasion step in primary site and the formation of metastasis in foreign organs. Additionally, exosomal miR-17-92a cluster extracted from patient serum was identified to correlate with high recurrence rate and poorer prognosis of CRC [55], which, from another perspective, confirmed our results that exosomal miR-17-5p was engaged in metastasis formation. However, our current results do not show the exact mechanism that how miR-17-5p is assembled into exosomes and how circLONP2 drives this process. Studies have shown that hnRNPA2B1 and other RBPs facilitate exosomal package of miRNAs through specific motifs [61]. We believe that miR-17-5p is assembled into exosomes through similar mechanism, which needs further investigations.

**Fig. 8:**
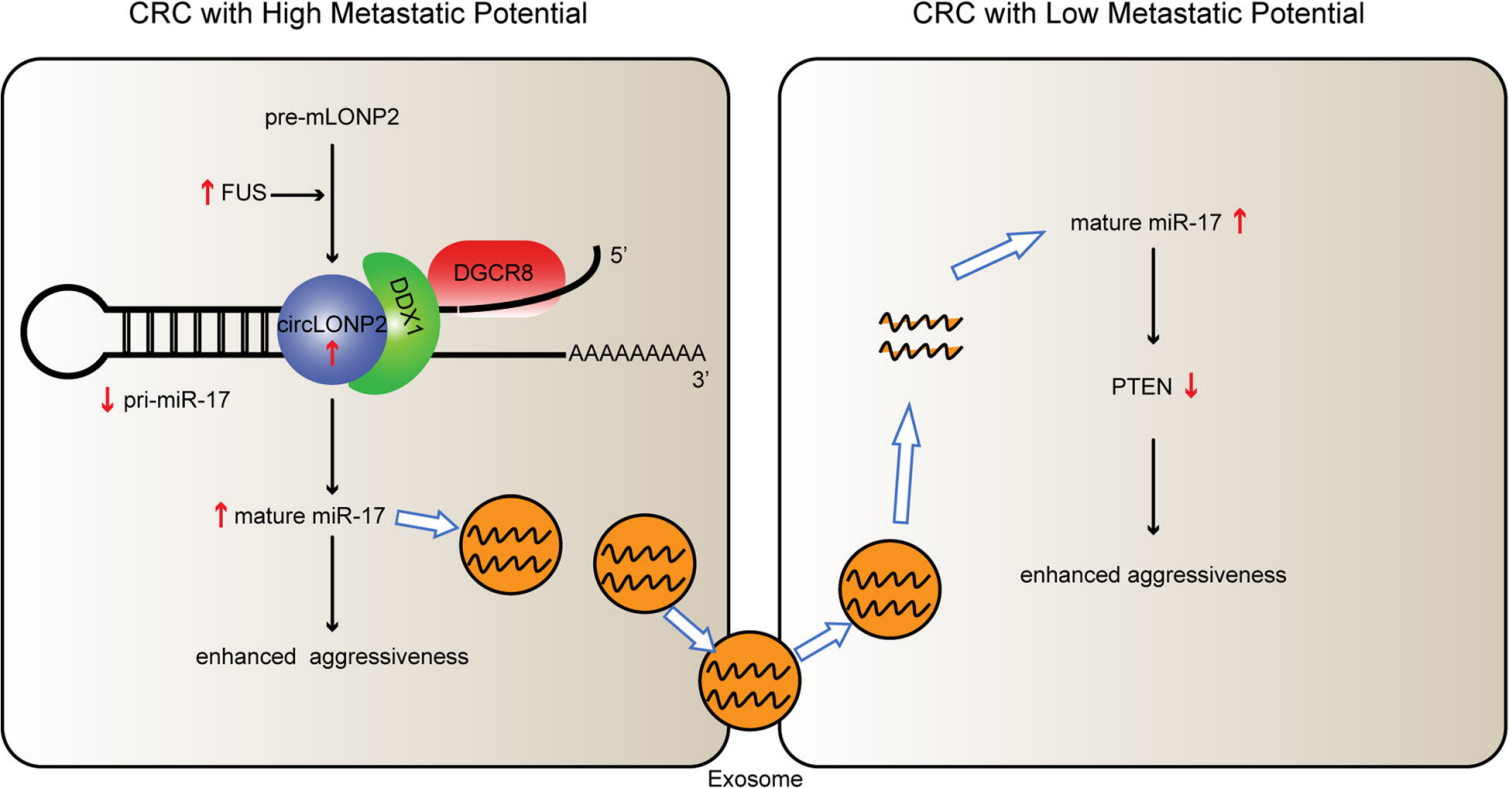
Function and mechanism of circLONP2 during CRC metastasis. circLONP2 acts as key metastasis-initiating molecule during CRC progression through modulating the intracellular maturation and intercellular transfer of miR-17, resulting in dissemination of metastasis-initiating ability and acceleration of metastasis formation

In summary, our results indicate that circLONP2 acts as key metastasis-initiating molecule in CRC progression through modulating the intracellular maturation and intercellular transfer of miR-17, resulting in dissemination of metastasis-initiating ability and acceleration of metastasis formation (Fig. 8). In particular, circLONP2-depletion greatly attenuates the initial homing of CRC cells to distant organs. Clinically, circLONP2 could serve as an effective prognostic predictor and/or novel anti-metastasis therapeutic target in CRC treatment.

## Supplementary information

**Additional file 1: Table S1.** Primers and siRNAs used in this article.

**Additional file 2: Table S2.** Univariate and multivariate Cox regression analysis of different prognostic variables in CRC patients.

**Additional file 3: Table S3.** Correlation analysis between circLONP2 expression and clinicopathological parameters of CRC.

**Additional file 4: Table S4.** Correlation analysis between FUS expression and clinicopathological parameters of CRC.

**Additional file 5: Table S5. **Deregulated miRNAs after circLONP2 overexpression or knockdown.

**Additional file 6: Figure S1.** Screening for CRC subgroups with different metastatic ability by Transwell assay. (A) Typical morphology of LM, HM subtype and the parent DLD-1 cells. (B) In vitro verification for the aggressiveness of LM and HM subtypes, respectively. (C) The proliferation ability of CRC subgroups tested by CCK8. (D, E) The EMT markers of CRC subgroups examined by WB. (F) Differentially expressed circRNAs in CRC subgroups showed by Venn diagram. (G) Verification of top 10 differentially expressed circRNAs in HM and normal parental CRC cell lines. (H) Sketch map for overexpressing plasmid of circLONP2 fused with GFP. (I, G) Image taken under microscope and anti-GFP WB indicated no GFP expression after transfected with the plasmid shown in (A), respectively. All experiments were repeated for three times, data were shown as mean ± SD, * *P* < 0.05, ** *P* < 0.01, *** *P* < 0.001, **** *P* < 0.0001 in independent Student’s t test (B), or two-way ANOVA (C).**Figure S2.** FUS regulates the biogenesis of circLONP2. (A) Highly matched RCMs in I7RCM and I11RCM. (B) Northern Blot demonstrated that the wild type with complete I7RCM and I11RCM could significantly overexpress circLONP2. (C) WB revealed significant knockdown of FUS by siRNA. (D) RNA binding motifs of FUS. (E) FUS protein was significantly increased in CRC tumor tissue (*n* = 4). **Figure S3.** Screening for potential proteins interacting with circLONP2. (A, B) The specific amino acid sequence of DDX5 and DDX17 showed by second-order mass spectrum, respectively. (C, D) Verification of DDX1 overexpression and knockdown by WB, respectively. (E, F) Rescue experiments indicated that DDX1 was essential for circLONP2-enhanced invasion ability of CRC cells. All experiments were repeated for three times, data were shown as mean±SD, * *P*<0.05, ** *P*<0.01, *** *P*<0.001, **** *P*<0.0001 in one-way ANOVA (E, F). **Figure S4.** circLONP2 and DDX1 collaboratively modulate pri-miR-17 processing. (A) Verification of pri-miR-17 and miR-17-3p/5p as downstream targets of DDX1 by RT-qPCR. (B) Detection efficiency of specific PCR primers for pri-miR-17 verified by DNase I treatment (C, D) Actinomycin D treatment revealed that circLONP2 could affect the RNA stability of pri-miR-17. (E) Potential interacting sequence between circLONP2 and pri-miR-17 showed by BLAST. (F) IP assay revealed that the interaction of DDX1 and DGCR8 did not depend on RNA. (G) RNA pulldown assay indicated that DDX1 mediated the interaction between circLONP2 and DGCR8. (H) Overexpressing DGCR8 or DDX1 could rescue the effect of ASO-circLONP2 on pri-miR-17. (I) Overexpression efficiency after transfected with miR-17-5p mimics, normalized to U6. (J) miR-17-5p was significantly increased in primary CRC tissues with advanced stage, normalized to U6. (K) The expression of miR-17-5p and circLNOP2 were significantly correlated in primary CRC tissues, normalized to U6 or GAPDH. (L) High expression of miR-17-5p was correlated with unfavorable prognosis of CRC patients. (M) The expression of miR-17-5p was significantly increased in liver metastasis than that in paired primary CRC tissues, normalized to U6. All experiments were repeated for three times, data were shown as mean ± SD, * *P* < 0.05, ** *P* < 0.01, *** *P* < 0.001, **** *P* < 0.0001 in one-way ANOVA (A, H), independent Student’s t test (B, J), two-way ANOVA (C, D), Mann-Whitney U test (I), person correlation test (K), log-rank test (L), or paired Student’s t test (M).**Figure S5.** Exosomal miR-17-5p disseminates high metastatic potential. (A) DLD-1 cells co-cultured with HM or circLONP2-overexpressed cells showed enhanced invasion ability, and this effect could be attenuated by GW4869 treatment. (B-D) Exosomes extracted from DLD-1 and DLD-1-HM cells were confirmed by electron microscopy, WB and NanoSight analysis, respectively. (E) Overexpression or knockdown of circLONP2 could increase or decrease the exosomal level of miR-17-5p, normalized to cel-miR-39. (F) The expression of RAB27A or RAB 27B was not changed upon circLONP2 overexpression or depletion. (G) CRC cells incubated with exosomes extracted from HM or circLONP2-overexpressed cells showed significantly enhanced migration ability. (E) The enhanced ability of receipt cells could sustain for at least 5 days after removal of exosomes. All experiments were repeated for three times, data were shown as mean ± SD, * *P* < 0.05, ** *P* < 0.01, *** *P* < 0.001, **** *P* < 0.0001 in one-way ANOVA (A, G), independent Student’s t test (E), or Mann-Whitney U test (H).

## Data Availability

The Gene Expression Omnibus accession numbers for miRNA microarray data are GSE121850 and GSE121851. The authenticity of this article has been validated by uploading the key raw data onto the Research Data Deposit public platform (www.researchdata.org.cn), with the approval RDD number as RDDB2020000813.

## Abbreviations

CRC: Colorectal carcinoma
miRNA: MicroRNA
circRNA: Circular RNA
RT-qPCR: Real time quantitative PCR
FISH: Fluorescence in situ hybridization
WB: Western blot
IP: Immunoprecipitation
RCMs: Reverse complementary matches
ASO: Anti-sense oligonucleotide
BLAST: Basic Local Alignment Search Tool
