# Correction to: CircLONP2 enhances colorectal carcinoma invasion and metastasis through modulating the maturation and exosomal dissemination of microRNA-17

**DOI:** 10.1186/s12943-021-01350-6

**Published:** 2021-03-31

**Authors:** Kai Han, Feng-Wei Wang, Chen-Hui Cao, Han Ling, Jie-Wei Chen, Ri-Xin Chen, Zi-Hao Feng, Jie Luo, Xiao-Han Jin, Jin-Ling Duan, Shu-Man Li, Ning-Fang Ma, Jing-Ping Yun, Xin-Yuan Guan, Zhi-Zhong Pan, Ping Lan, Rui-Hua Xu, Dan Xie

**Affiliations:** 1 Sun Yat-sen University Cancer Center; State Key Laboratory of Oncology in South China, Collaborative Innovation Center for Cancer Medicine, Guangzhou, China; 2 Department of Colorectal Surgery, Sun Yat-sen University Cancer Center, Guangzhou, China; 3 Department of Pathology, Sun Yat-sen University Cancer Center, Guangzhou, China; 4 Department of Surgery, First Affiliated Hospital, Sun Yat-sen University, Guangzhou, China; 5 Key Laboratory of Protein Modification and Degradation, School of Basic Medical Sciences, Affiliated Cancer Hospital & Institute of Guangzhou Medical University, Guangzhou, China; 6 Department of Clinical Oncology, The University of Hong Kong, Hong Kong, China; 7 Department of Colorectal Surgery, The Six Affiliated Hospital, Sun Yat-sen University, Guangzhou, China

**Correction to: Mol Cancer (2020) 19:60**

**https://doi.org/10.1186/s12943-020-01184-8**

Following publication of the original article [1], the authors identified some minor errors in image-typesetting in Fig. 4; specifically, the transwell invasion assay of HCT116 cells with circLONP2-overexpression shown in Fig. 4b.

**Fig. 4:**
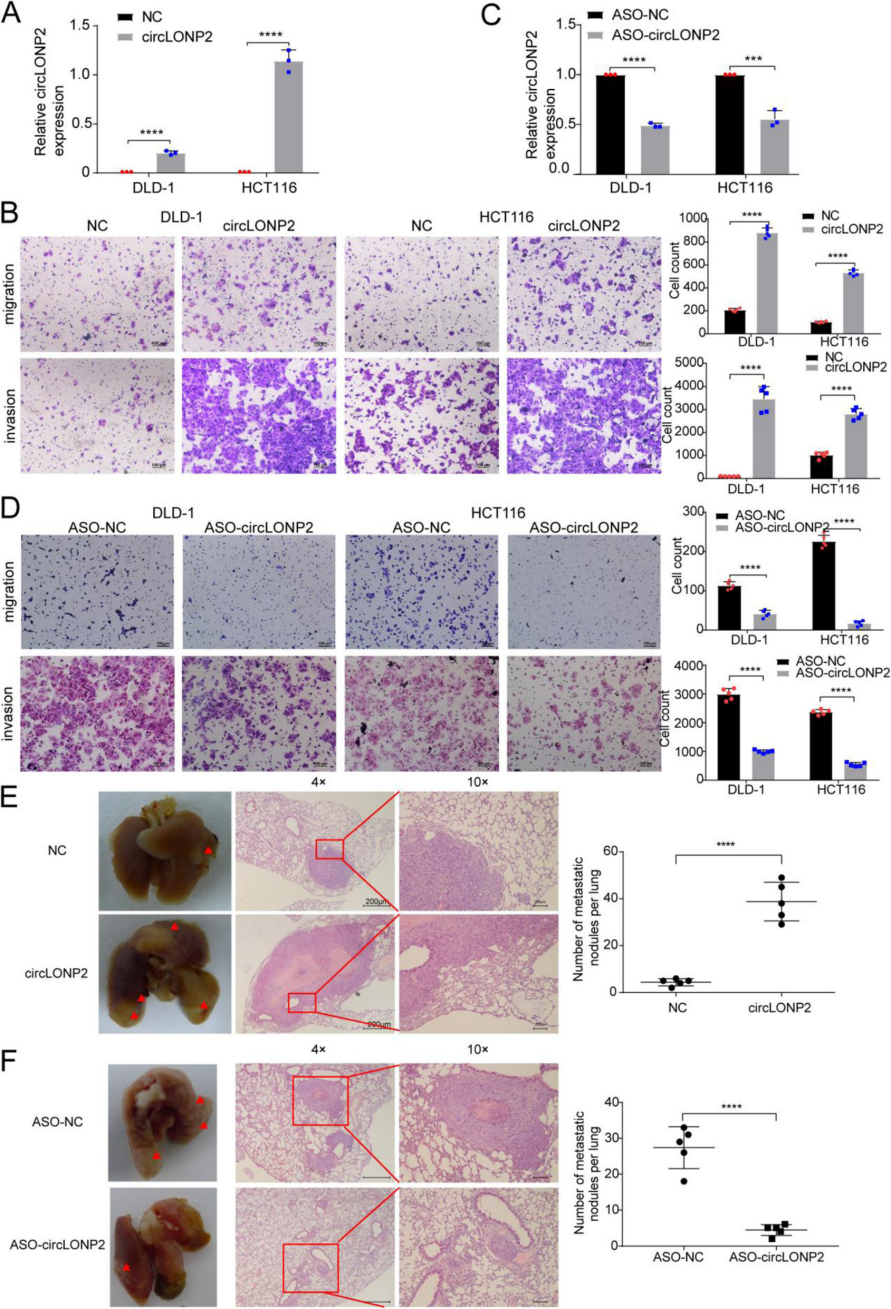
circLONP2 is essential for CRC metastasis. **a**, **b** Overexpression of circLONP2 significantly enhanced the migration and invasion ability of CRC cells. **c**, **d** Knockdown of circLONP2 by ASO significantly suppressed the migration and invasion ability of CRC cells. **e**, **f** In vivo tail vein injection model confirmed that overexpression or knockdown of circLONP2 could significantly promote or attenuate CRC cells metastasize to lung, respectively. All detection of circLONP2 by RT-qPCR was normalized to GAPDH. All experiments were repeated for three times, data were shown as mean±SD, * *P*<0.05, ** *P*<0.01, *** *P*<0.001, **** *P*<0.0001 in Mann-Whitney U test (**a**, **c**, **e**, **f**), or independent Student’s t test (**b**, **d**)

The corrected figure is given below. The corrections do not have any effect on the final conclusions of the paper.
