# Supplementary material for: CircLONP2 enhances colorectal carcinoma invasion and metastasis through modulating the maturation and exosomal dissemination of microRNA-17
Source: Mol Cancer. 2020 Mar 18;19:60. doi: 10.1186/s12943-020-01184-8 (PMC7079398; doi:10.1186/s12943-020-01184-8)
Supplement: Supplementary file 2 — Additional file 2: Table S2. Univariate and multivariate Cox regression analysis of different prognostic variables in CRC patients. [file 12943_2020_1184_MOESM2_ESM.docx]

| Variable | Subset | Hazard ratio for OS (95% CI) | *P value* |
| --- | --- | --- | --- |
| Univariate analysis (n=128) | | | |
| Age (yr) | <60 vs ≥60 | - | 0.179 |
| Gender | Male vs Femal | - | 0.206 |
| pT status | T1+2 vs T3+4 | - | 0.770 |
| pN status | N0 vs N1 | 0.037 (0.011-0.122) | <0.001 |
| pM status | M0 vs M1 | 0.106 (0.042-0.269) | <0.001 |
| Clinical stage | I+II vs III+IV | 0.042 (0.011-0.159) | <0.001 |
| circLONP2 expression | Low vs High | 0.399 (0.156-1.017) | 0.054 |
| Multivariate analysis (n=128) | | | |
| pN status | N0 vs N1 | 42.545 (11.240-161.037) | <0.001 |
| pM status | M0 vs M1 | 12.385 (4.029-38.071) | <0.001 |
| Clinical stage | I+II vs III+IV | - | 0.515 |
| circLONP2 expression | Low vs High | 0.259 (0.081-0.826) | 0.022 |

**Table S2 Univariate and multivariate** **Cox regression analysis of different prognostic variables in CRC patients**
