# Supplementary material for: CircLONP2 enhances colorectal carcinoma invasion and metastasis through modulating the maturation and exosomal dissemination of microRNA-17
Source: Mol Cancer. 2020 Mar 18;19:60. doi: 10.1186/s12943-020-01184-8 (PMC7079398; doi:10.1186/s12943-020-01184-8)
Supplement: Supplementary file 3 — Additional file 3: Table S3. Correlation analysis between circLONP2 expression and clinicopathological parameters of CRC. [file 12943_2020_1184_MOESM3_ESM.doc]

**Table S3 Correlation analysis between circLONP2 expression and clinicopathological parameters of CRC**

| Variable | Number of cases | circLONP2 expression | | | *P** |
| --- | --- | --- | --- | --- | --- |
|  |  | High expression (*N*=64) | Low expression (*N*=64) | |  |
| Age, yr |  |  | |  |  |
| ≥60 | 54 | 23 | | 31 |  |
| <60 | 74 | 41 | | 33 | 0.152 |
| Gender |  |  | |  |  |
| Female | 54 | 24 | | 30 |  |
| Male | 74 | 40 | | 34 | 0.283 |
| Tumour location |  |  | |  |  |
| Colon | 72 | 39 | | 33 |  |
| Rectum | 56 | 25 | | 31 | 0.285 |
| pT status |  |  | |  |  |
| T1-T2 | 16 | 7 | | 9 |  |
| T3-T4 | 112 | 57 | | 55 | 0.592 |
| pN status |  |  | |  |  |
| N0 | 81 | 35 | | 46 |  |
| N1-N2 | 47 | 29 | | 18 | 0.044 |
| pM status |  |  | |  |  |
| M0 | 100 | 45 | | 55 |  |
| M1 | 28 | 19 | | 9 | 0.033 |
| Clinical stage |  |  | |  |  |
| I+II | 69 | 28 | | 41 |  |
| III+IV | 59 | 36 | | 23 | 0.021 |

CRC, colorectal carcinoma; * χ^2^ text
