# Supplementary material for: CircLONP2 enhances colorectal carcinoma invasion and metastasis through modulating the maturation and exosomal dissemination of microRNA-17
Source: Mol Cancer. 2020 Mar 18;19:60. doi: 10.1186/s12943-020-01184-8 (PMC7079398; doi:10.1186/s12943-020-01184-8)
Supplement: Supplementary file 4 — Additional file 4: Table S4. Correlation analysis between FUS expression and clinicopathological parameters of CRC. [file 12943_2020_1184_MOESM4_ESM.docx]

**Table S4 Correlation analysis between FUS expression and clinicopathological parameters of CRC**

| Variable | Number of cases | FUS expression | | | *P** |
| --- | --- | --- | --- | --- | --- |
|  | N=110 | High expression (*N*=55) | Low expression (*N*=55) | |  |
| Age, yr |  |  | |  | 0.445 |
| ≥60 | 52 | 28 | | 24 |  |
| <60 | 58 | 27 | | 31 |  |
| Gender |  |  | |  | 0.178 |
| Female | 49 | 24 | | 22 |  |
| Male | 61 | 31 | | 33 |  |
| Tumour location |  |  | |  | 0.432 |
| Colon | 68 | 36 | | 32 |  |
| Rectum | 42 | 19 | | 23 |  |
| pT status |  |  | |  | 0.567 |
| T1-T2 | 14 | 6 | | 8 |  |
| T3-T4 | 96 | 49 | | 47 |  |
| pN status |  |  | |  | 0.028 |
| N0 | 71 | 30 | | 41 |  |
| N1-N2 | 39 | 25 | | 14 |  |
| pM status |  |  | |  | 0.000 |
| M0 | 85 | 36 | | 49 |  |
| M1 | 25 | 19 | | 6 |  |
| Clinical stage |  |  | |  | 0.004 |
| I+II | 63 | 24 | | 39 |  |
| III+IV | 47 | 31 | | 16 |  |

CRC, colorectal carcinoma; * χ^2^ text
