# Supplementary material for: CircLONP2 enhances colorectal carcinoma invasion and metastasis through modulating the maturation and exosomal dissemination of microRNA-17
Source: Mol Cancer. 2020 Mar 18;19:60. doi: 10.1186/s12943-020-01184-8 (PMC7079398; doi:10.1186/s12943-020-01184-8)
Supplement: Supplementary file 6 — Additional file 6: Figure S1. Screening for CRC subgroups with different metastatic ability by Transwell assay. (A) Typical morphology of LM, HM subtype and the parent DLD-1 cells. (B) In vitro verification for the aggressiveness of LM and HM subtypes, respectively. (C) The proliferation ability of CRC subgroups tested by CCK8. (D, E) The EMT markers of CRC subgroups examined by WB. (F) Differentially expressed circRNAs in CRC subgroups showed by Venn diagram. (G) Verification of top 10 differentially expressed circRNAs in HM and normal parental CRC cell lines. (H) Sketch map for overexpressing plasmid of circLONP2 fused with GFP. (I, G) Image taken under microscope and anti-GFP WB indicated no GFP expression after transfected with the plasmid shown in (A), respectively. All experiments were repeated for three times, data were shown as mean ± SD, * P < 0.05, ** P < 0.01, *** P < 0.001, **** P < 0.0001 in independent Student’s t test (B), or two-way ANOVA (C).Figure S2. FUS regulates the biogenesis of circLONP2. (A) Highly matched RCMs in I7RCM and I11RCM. (B) Northern Blot demonstrated that the wild type with complete I7RCM and I11RCM could significantly overexpress circLONP2. (C) WB revealed significant knockdown of FUS by siRNA. (D) RNA binding motifs of FUS. (E) FUS protein was significantly increased in CRC tumor tissue (n = 4). Figure S3. Screening for potential proteins interacting with circLONP2. (A, B) The specific amino acid sequence of DDX5 and DDX17 showed by second-order mass spectrum, respectively. (C, D) Verification of DDX1 overexpression and knockdown by WB, respectively. (E, F) Rescue experiments indicated that DDX1 was essential for circLONP2-enhanced invasion ability of CRC cells. All experiments were repeated for three times, data were shown as mean±SD, * P<0.05, ** P<0.01, *** P<0.001, **** P<0.0001 in one-way ANOVA (E, F). Figure S4. circLONP2 and DDX1 collaboratively modulate pri-miR-17 processing. (A) Verification of pri-miR-17 and miR-17-3p/ [file 12943_2020_1184_MOESM6_ESM.docx]

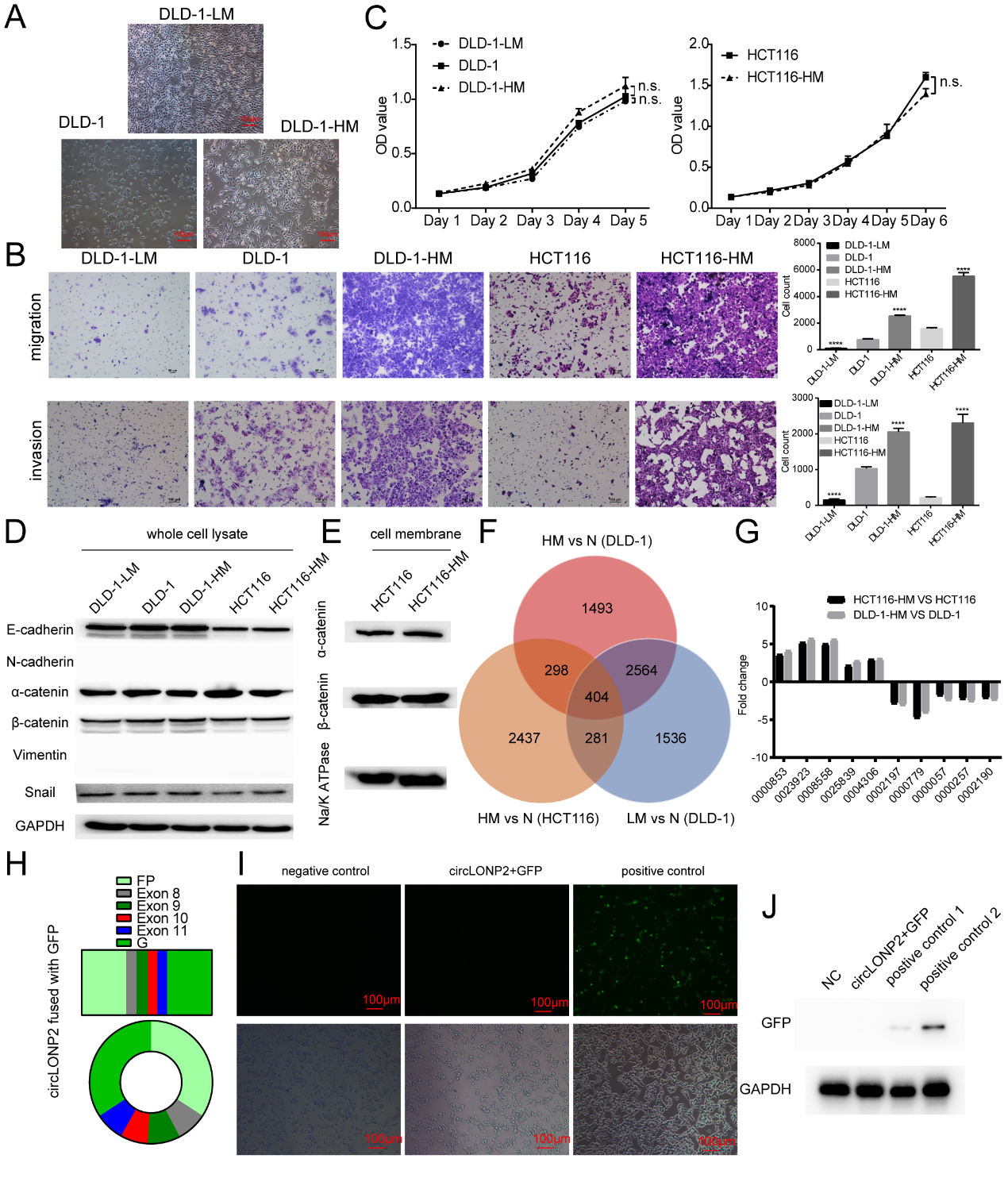


**Fig. S1 Screening for CRC subgroups with different metastatic ability by Transwell assay.** (A) Typical morphology of LM, HM subtype and the parent DLD-1 cells. (B) *In vitro* verification for the aggressiveness of LM and HM subtypes, respectively. (C) The proliferation ability of CRC subgroups tested by CCK8. (D, E) The EMT markers of CRC subgroups examined by WB. (F) Differentially expressed circRNAs in CRC subgroups showed by Venn diagram. (G) Verification of top 10 differentially expressed circRNAs in HM and normal parental CRC cell lines. (H) Sketch map for overexpressing plasmid of circLONP2 fused with GFP. (I, G) Image taken under microscope and anti-GFP WB indicated no GFP expression after transfected with the plasmid shown in (A), respectively. All experiments were repeated for three times, data were shown as mean±SD, * P<0.05, ** P<0.01, *** P<0.001, **** P<0.0001 in independent Student’s t test (B), or two-way ANOVA (C).


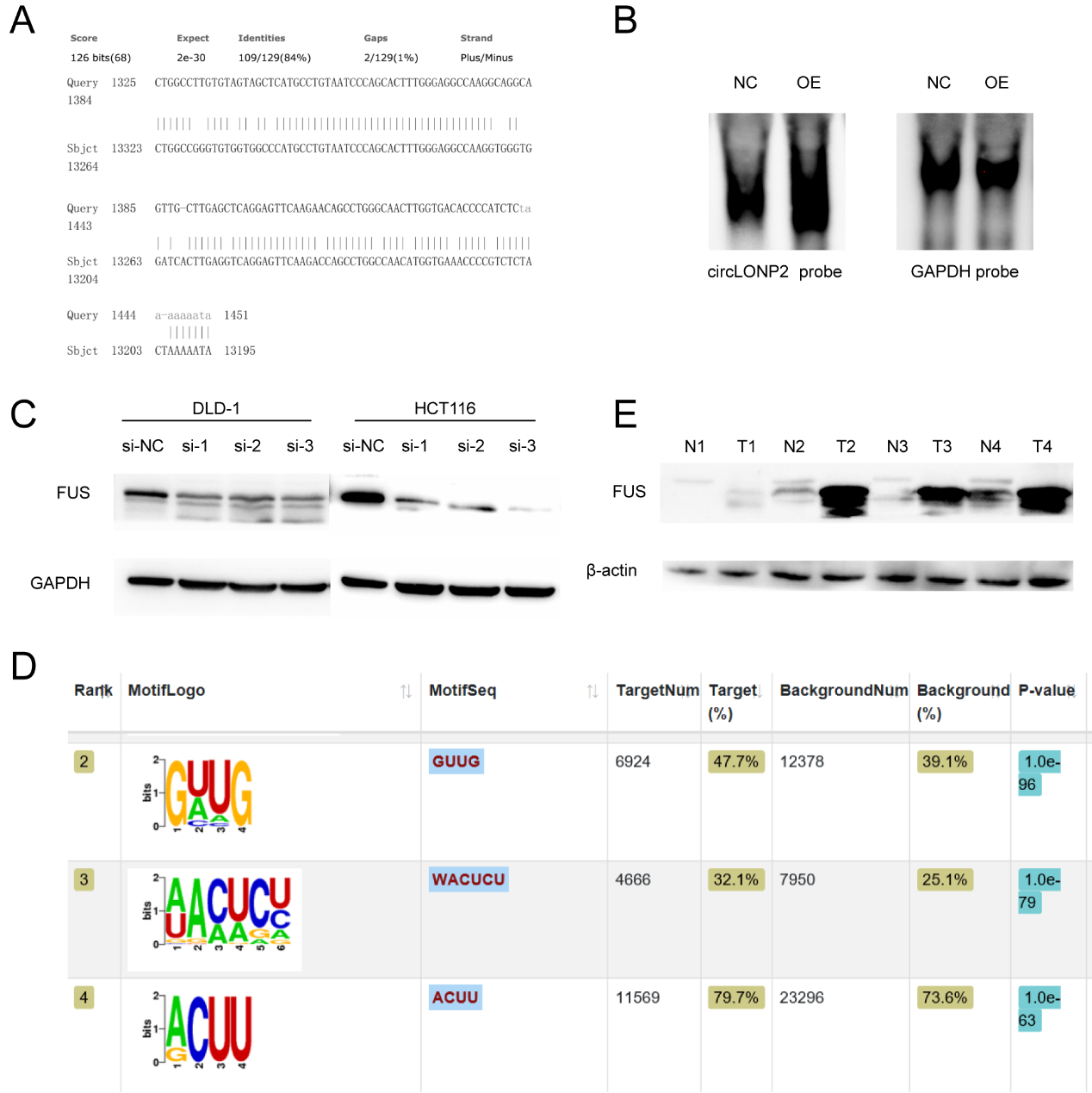


**Fig. S2 FUS regulates the biogenesis of circLONP2.** (A) Highly matched RCMs in I7RCM and I11RCM. (B) Northern Blot demonstrated that the wild type with complete I7RCM and I11RCM could significantly overexpress circLONP2. (C) WB revealed significant knockdown of FUS by siRNA. (D) RNA binding motifs of FUS. (E) FUS protein was significantly increased in CRC tumor tissue (n=4).


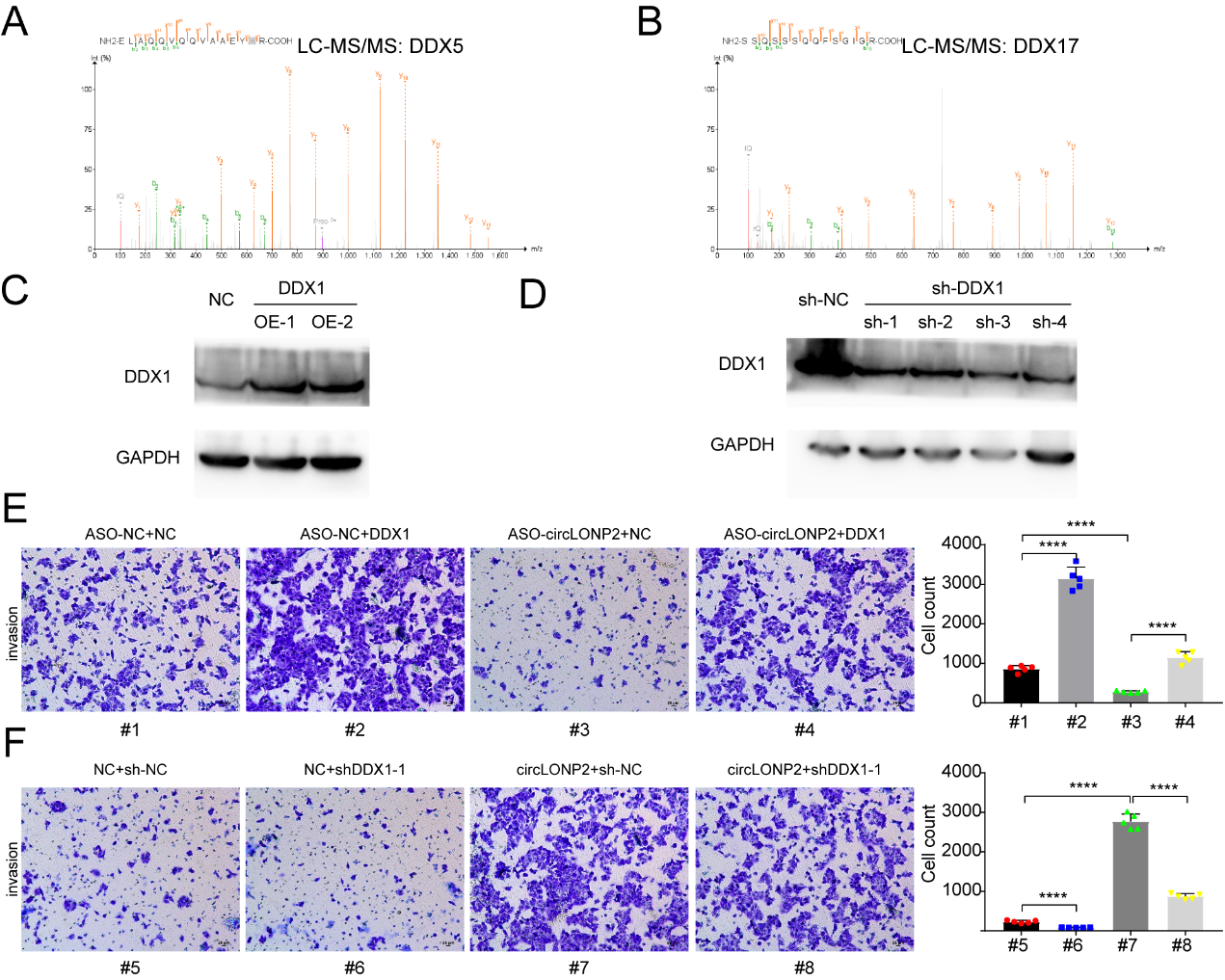


**Fig. S3 Screening for potential proteins interacting with circLONP2.** (A, B) The specific amino acid sequence of DDX5 and DDX17 showed by second-order mass spectrum, respectively. (C, D) Verification of DDX1 overexpression and knockdown by WB, respectively. (E, F) Rescue experiments indicated that DDX1 was essential for circLONP2-enhanced invasion ability of CRC cells. All experiments were repeated for three times, data were shown as mean±SD, * P<0.05, ** P<0.01, *** P<0.001, **** P<0.0001 in one-way ANOVA (E, F).


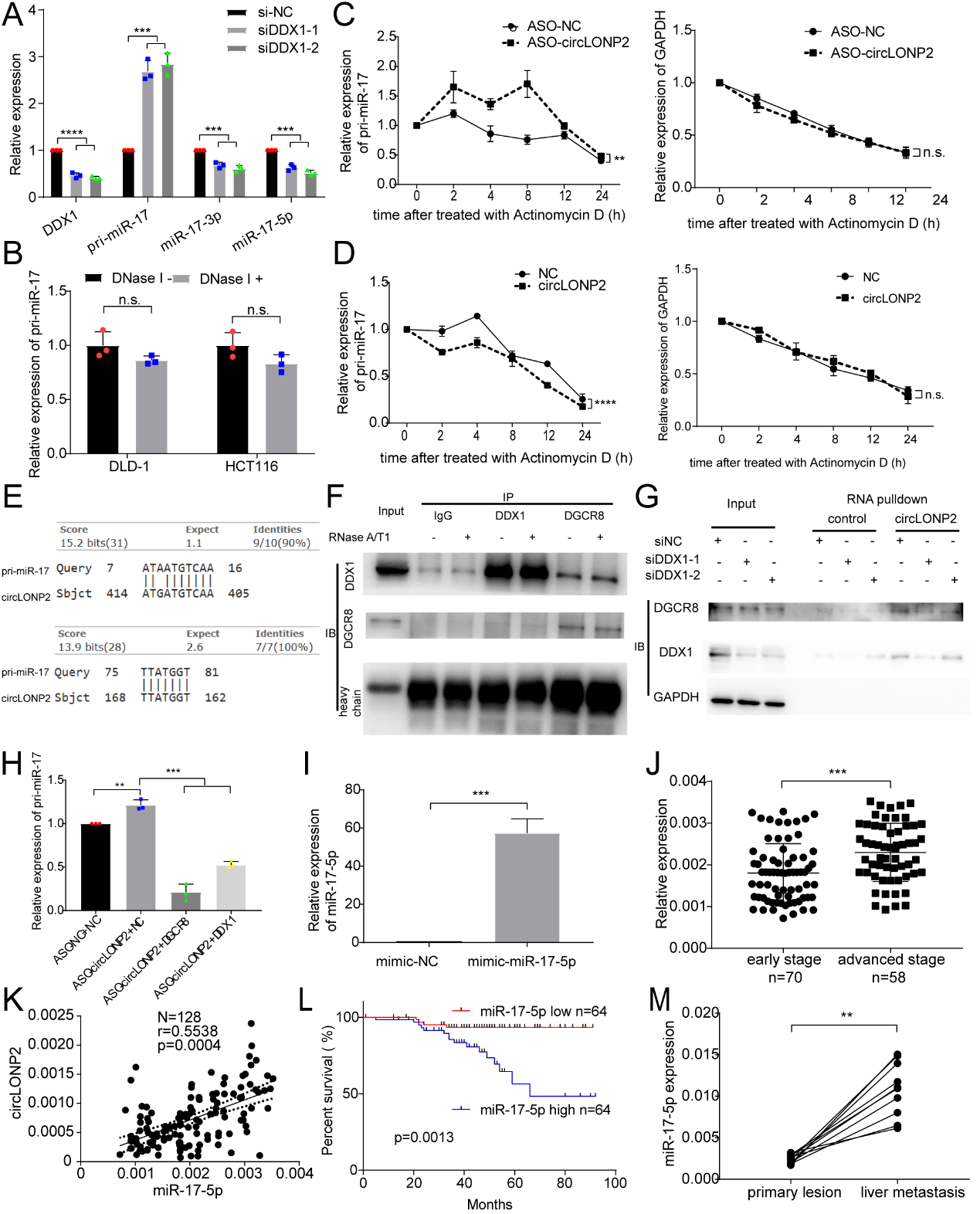


**Fig. S4** **circLONP2 and DDX1 collaboratively modulate pri-miR-17 processing.** (A) Verification of pri-miR-17 and miR-17-3p/5p as downstream targets of DDX1 by RT-qPCR. (B) Detection efficiency of specific PCR primers for pri-miR-17 verified by DNase I treatment (C, D) Actinomycin D treatment revealed that circLONP2 could affect the RNA stability of pri-miR-17. (E) Potential interacting sequence between circLONP2 and pri-miR-17 showed by BLAST. (F) IP assay revealed that the interaction of DDX1 and DGCR8 did not depend on RNA. (G) RNA pulldown assay indicated that DDX1 mediated the interaction between circLONP2 and DGCR8. (H) Overexpressing DGCR8 or DDX1 could rescue the effect of ASO-circLONP2 on pri-miR-17. (I) Overexpression efficiency after transfected with miR-17-5p mimics, normalized to U6. (J) miR-17-5p was significantly increased in primary CRC tissues with advanced stage, normalized to U6. (K) The expression of miR-17-5p and circLNOP2 were significantly correlated in primary CRC tissues, normalized to U6 or GAPDH. (L) High expression of miR-17-5p was correlated with unfavorable prognosis of CRC patients. (M) The expression of miR-17-5p was significantly increased in liver metastasis than that in paired primary CRC tissues, normalized to U6. All experiments were repeated for three times, data were shown as mean±SD, * P<0.05, ** P<0.01, *** P<0.001, **** P<0.0001 in one-way ANOVA (A, H), independent Student’s t test (B, J), two-way ANOVA (C, D), Mann-Whitney U test (I), person correlation test (K), log-rank test (L), or paired Student’s t test (M).

**
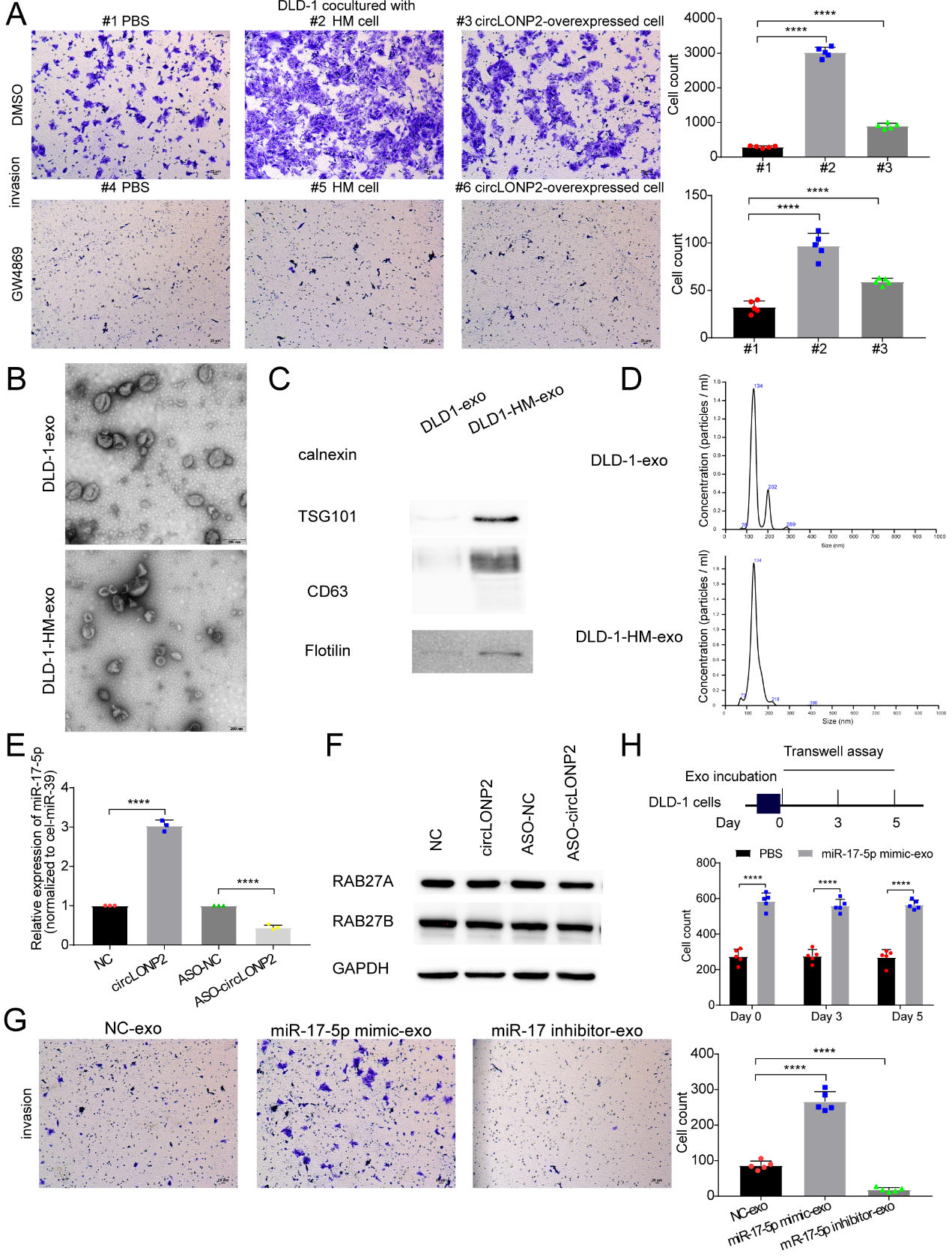
**

**Fig. S5 Exosomal miR-17-5p disseminates high metastatic potential.** (A) DLD-1 cells co-cultured with HM or circLONP2-overexpressed cells showed enhanced invasion ability, and this effect could be attenuated by GW4869 treatment. (B-D) Exosomes extracted from DLD-1 and DLD-1-HM cells were confirmed by electron microscopy, WB and NanoSight analysis, respectively. (E) Overexpression or knockdown of circLONP2 could increase or decrease the exosomal level of miR-17-5p, normalized to cel-miR-39. (F) The expression of RAB27A or RAB 27B was not changed upon circLONP2 overexpression or depletion. (G) CRC cells incubated with exosomes extracted from HM or circLONP2-overexpressed cells showed significantly enhanced migration ability. (E) The enhanced ability of receipt cells could sustain for at least 5 days after removal of exosomes. All experiments were repeated for three times, data were shown as mean±SD, * P<0.05, ** P<0.01, *** P<0.001, **** P<0.0001 in one-way ANOVA (A, G), independent Student’s t test (E), or Mann-Whitney U test (H).
